# A diverse uncultivated microbial community is responsible for organic matter degradation in the Black Sea sulphidic zone

**DOI:** 10.1111/1462-2920.14902

**Published:** 2020-01-13

**Authors:** Saara Suominen, Nina Dombrowski, Jaap S. Sinninghe Damsté, Laura Villanueva

**Affiliations:** ^1^ Department of Marine Microbiology and Biogeochemistry NIOZ Royal Netherlands Institute for Sea Research and Utrecht University Den Hoorn The Netherlands; ^2^ Department of Earth Sciences, Faculty of Geosciences Utrecht University Utrecht The Netherlands

## Abstract

Organic matter degradation in marine environments is essential for the recycling of nutrients, especially under conditions of anoxia where organic matter tends to accumulate. However, little is known about the diversity of the microbial communities responsible for the mineralization of organic matter in the absence of oxygen, as well as the factors controlling their activities. Here, we determined the active heterotrophic prokaryotic community in the sulphidic water column of the Black Sea, an ideal model system, where a tight coupling between carbon, nitrogen and sulphur cycles is expected. Active microorganisms degrading both dissolved organic matter (DOM) and protein extracts were determined using quantitative DNA stable isotope probing incubation experiments. These results were compared with the metabolic potential of metagenome‐assembled genomes obtained from the water column. Organic matter incubations showed that groups like *Cloacimonetes* and *Marinimicrobia* are generalists degrading DOM. Based on metagenomic profiles the degradation proceeds in a potential interaction with members of the *Deltaproteobacteria* and *Chloroflexi Dehalococcoidia*. On the other hand, microbes with small genomes like the bacterial phyla *Parcubacteria*, *Omnitrophica* and of the archaeal phylum *Woesearchaeota*, were the most active, especially in protein‐amended incubations, revealing the potential advantage of streamlined microorganisms in highly reduced conditions.

## Introduction

Organic matter (OM) turnover in the oceans is driven by microbial activity but very little is known of the organisms that are performing this process in anoxic environments. Niches in oxygen‐depleted environments have traditionally been defined by available electron sinks (reviewed in Ulloa *et al*., 2012; Wright *et al*., 2012; Bertagnolli and Stewart, 2012), while the role of carbon sources in shaping these communities has not often been addressed. Since sinking OM sustains the microbial communities in the water column (Pomeroy, 1974; Azam *et al*., 1983, Azam, 1998), the adaptation to degrade different organic molecules is possibly one of the major factors defining the niches of water column organisms (Cottrell and Kirchman, 2000; Kujawinski, 2011; Gomez‐Consarnau *et al*., 2012).

The advent of sequencing methods and genomic approaches are expanding our understanding of the bacterial and archaeal phyla that are predicted, based on their genome, to be involved in OM processing in the absence of oxygen (Wrighton *et al*., 2012; Brown *et al*., 2015; Castelle *et al*., 2015, 2018; Hug *et al*., 2016; Solden *et al*., 2016). The taxonomic groups to which these organisms belong to form a major part of all detected sequences in natural environments (Lloyd *et al*., 2018). However, these organisms often resist traditional cultivation methods, which could be attributed to their lifestyle in highly connected and co‐dependent microbial communities, and, therefore, novel cultivation‐independent approaches are necessary to describe the functioning of these microbial communities. While (meta)genomics and transcriptomics data can give us an estimation of the potential capabilities of microbial communities and their uncultivated members, the direct observation of activity in OM degradation processes is necessary to understand the relationships and interactions of microbial cells with each other. Methods like DNA‐stable isotope probing (DNA‐SIP) allows us to track the metabolic flow of a substrate through a microbial community and increases our ability to understand the activity of microorganisms *in situ*. In addition, the combination of DNA‐SIP with the analytical resolution provided by next‐generation sequencing methodologies provides an opportunity to define active microbial members from complex communities (Bell *et al*., 2011; Hungate *et al*., 2015; Orsi *et al*., 2016; Pepe‐Ranney *et al*., 2016). For example, previous DNA‐SIP studies have given insights on the anaerobic heterotrophic carbon utilization in intertidal sediments (Webster *et al*., 2010; Graue *et al*., 2011, 2012), freshwater sediments (Coskun *et al*., 2018), soils (Pepe‐Ranney *et al*., 2016), salt marsh sediments (Seyler *et al*., 2014), and sands (Gihring *et al*., 2009). For water columns, DNA‐SIP has so far been used to evaluate the degradation of protein and high molecular weight dissolved organic nitrogen only in oxic and hypoxic conditions (Orsi *et al*., 2016; Liu *et al*., 2017).

Here, we focused on the microbial community of the anoxic and sulphide‐containing (euxinic) water column of the Black Sea, which is the largest stable anoxic basin in the world. It contains a shallow oxic layer of about 100 m, which overlays sulphidic waters spanning 2000 m in depth (Oguz *et al*., 2004). OM is preferentially preserved in euxinic conditions. This preservation is hypothesized to be due to the inability of organisms to degrade recalcitrant organic molecules in the absence of oxygen (Canfield, 1989, 1994; Bastviken *et al*., 2004). However, net dissolved organic carbon (DOC) removal (Margolin *et al*., 2016), as well as detected high carboxylic acid and amino acid concentrations (Mopper and Kieber, 1991; Albert *et al*., 1995), point to active anaerobic carbon degradation processes. This makes it probable that the unknown microbial community in the Black Sea water column relies on labile OM for their metabolic needs. In addition, there is evidence that the degradation of nitrogenous compounds is preferred when oxygen is depleted (Van Mooy *et al*., 2002; Pantoja *et al*., 2004, 2009; Engel *et al*., 2017), making organic nitrogen compounds (e.g. proteins) a potentially important substrate type.

In this study, we identify OM‐metabolizing microbial communities in the euxinic Black Sea. Specifically, we examined the changes in the microbial diversity and metabolic preference of the active anaerobic heterotrophic microbial community in anoxic sulphidic waters (at 1000 m depth) by performing incubations with two complex carbon substrates: ^13^C‐ and ^15^N‐labelled dissolved organic matter (DOM) and protein extracts. Additionally, we compared the activity of specific microbial groups with the metabolic potential of phylogenetically related metagenome‐assembled genomes (MAGs) obtained from the water column.

## Results

### Physicochemical conditions and microbial community composition at 1000 m depth

**Fig. 1:**
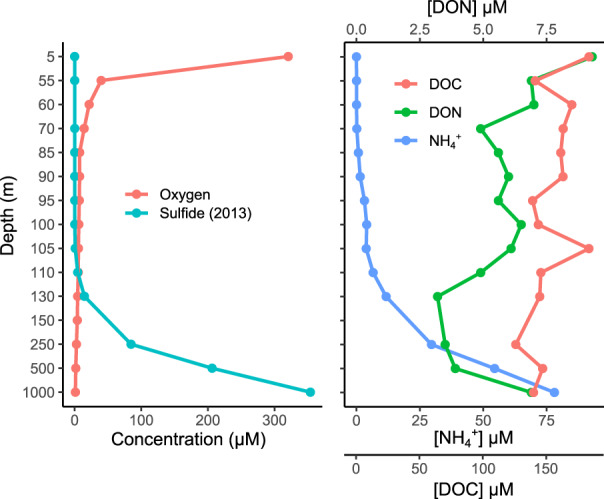
Water column physicochemical measurements at the sampling site. DON: dissolved organic nitrogen, DOC: dissolved organic carbon. Note that the *y*‐axis is not to scale. [Color figure can be viewed at wileyonlinelibrary.com]

At the time of sampling, the Black Sea water column was fully anoxic starting from 100 m depth (Fig. 1). The sulphide concentration was measured during the cruise in 2013; it reached a maximum of 400 μM at 2000 m depth (Sollai *et al*., 2018). There was also an accumulation of ammonium reaching approximately 80 μM in the deep waters (Fig. 1). DOC and dissolved organic DON were 140 and 6.91 μM respectively, at 1000 m.

**Fig. 2:**
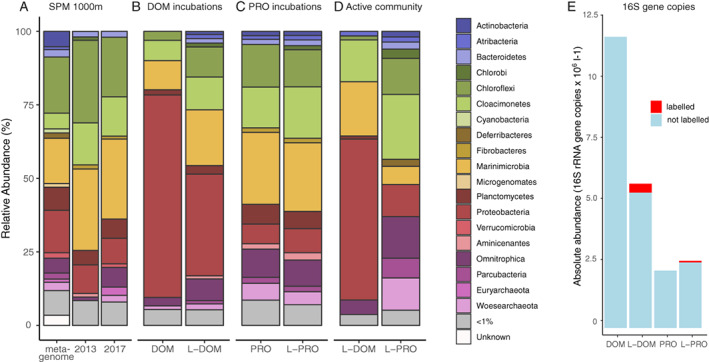
Prokaryotic community composition shown as the relative abundance of 16S rRNA genes in the Black Sea water column at 1000 m with and without the addition of OM.A. Water column community from collected suspended particulate matter (SPM) using the metagenomic data, 2013 and 2017 amplicon data sets.B. Incubations with unlabelled dissolved organic matter (DOM) and ^13^C/^15^N labelled dissolved organic matter (L‐DOM).C. Incubations with unlabelled protein (PRO) and ^13^C/^15^N labelled protein (L‐PRO).D. The community composition of the active operational taxonomic units (OTUs). E. Absolute abundance of prokaryotes (estimated as 16S rRNA gene copies L^−1^) for the different treatments, with red areas depicting the amount of copies that were assigned to labelled OTUs calculated by using the relative abundance of OTUs assigned as active. [Color figure can be viewed at wileyonlinelibrary.com]

The prokaryotic community composition of the anoxic water at 1000 m was analysed in both 2013 and 2017 with 16S rRNA gene amplicon sequencing (Fig. 2A). In addition, the taxonomic composition of the 16S rRNA genes extracted from the metagenomes in 2013 (see below) was also analysed to estimate possible biases arising from PCR amplification (Fig. 2A). In the amplicon data sets, the only difference between the years was the filter size used for sample collection (0.7 μm‐pore size glass fibre filter and 0.22 μm‐pore size polycarbonate filter respectively). Despite the different pore size filters used, the prokaryotic community composition was similar in both years (Fig. 2A). The most abundant bacterial taxonomic groups present belonged to the phylum *Marinimicrobia* (28% in 2013% and 27% in 2017 respectively). *Chloroflexi* were the next most abundant phylum (28% and 20%), mainly belonging to the genus *Thermoflexus* (12% and 15%). Members of the phylum *Cloacimonetes* comprised approximately 14% and 13% of the total community and were mostly comprised of the class MSBL2. Other abundant groups were *Deltaproteobacteria* (9% and 8%), belonging predominantly to the genus *Desulfatigans* (4.2% and 4.3%) and the uncultured SEEP‐SRB1 group (3.7% and 3.2%), and the phylum *Planctomycetes* (4.9% and 6.5%), which were dominated by the candidate order MSBL9 in both years (1.7% and 2.2%), and *Phycisphaerales* in 2017 (2.3%; not detected in 2013). Similarly, the phylum *Omnitrophica* was more abundant in 2017 (1.1% and 6.6%). Other phyla comprising >1% of the total community were *Bacteroidetes* (1.8% and 2%), *Aminicenantes* (1.2% and 0.2%) and *Chlorobi* (1.1% and 0.6%).

The most abundant archaeal community members belonged to the anaerobic methane‐oxidizing archaea ANME‐1b within the *Euryarchaeota* (<1% in 2013, 2.4% in 2017), as well as to the phylum *Woesearchaeota* (<1% and 2.2%) belonging to the DPANN (*Diapherotrites*, *Parvarchaeota*, *Aenigmarchaeota*, *Nanoarchaeota* and *Nanohaloarchaeota*) superphylum.

### Microbial community composition after incubations with DOM and protein extracts

DNA‐SIP experiments were conducted with ^13^C and ^15^N‐labelled and unlabelled DOM and protein extracts, in a total of four incubations lasting 72 h (Fig. S2). We used a labile carbon source as a proxy for the degradation of compounds from different biochemical categories, which does not reflect the natural OM available at 1000 m in the Black Sea. The analyses resulted in the identification of 15 279 individual operational taxonomic units (OTUs). Compared to the natural microbial community composition, incubations with labelled and unlabelled DOM resulted in an increase from 0.04% (average in 2013 and 2017) to 28%–66% of the relative abundance of 16S rRNA gene sequences closely related to the *Gammaproteobacteria* genus *Psychromonas* (Fig. 2B). Other abundant community members belonged to the phyla *Marinimicrobia* (10% and 19%), *Cloacimonetes* (6.8% and 11%), *Thermoflexales* from the phylum *Chloroflexi* (3.1% and 3.3%) and members of the phylum *Omnitrophica* (2.9% and 7.3%) (Fig. 2B). However, their relative abundance was slightly lower than in the natural situation because of the huge increase of *Psychromonas* spp.

In the incubations with unlabelled and labelled protein, the microbial community composition differed less from the natural situation (Fig. 2C); the most common phylum was *Marinimicrobia* (SAR406) (24% and 23% respectively), followed by *Cloacimonetes* (13% and 17%) and members of *Chloroflexi* (15% and 13%), mainly from the order *Thermoflexales* (5.5% and 3.8%) and from the candidate order GIF9 (1.2% and 2.0%) (Fig. 2C).

The total community composition clearly differed between the two substrates added. In the incubations with labelled substrates, 45% of the OTUs were shared between the two different substrate incubations. The taxonomic composition of the labelled and unlabelled incubations for each substrate was more similar than when those for the different substrates were compared (cf. Fig. 2B and C).

### Microbial community composition with ^13^C and ^15^N labelled substrates

**Fig. 3:**
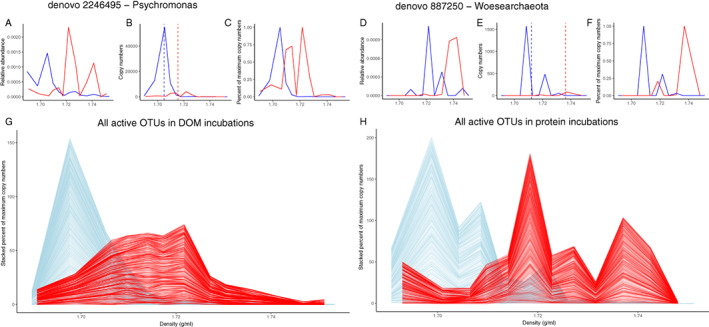
Comparison of the distributions of two individual and all (stacked) active OTUs across the density gradient in the qSIP experiments for the unlabelled and labelled incubations. Panels A–C and D–F show two examples (i.e. *Psychromonas* and *Woesearcheaota* OTUs respectively) of the results obtained during qSIP calculations aimed to determine labelled OTUs. Panels A and D show the relative abundance (in fractional abundance) of the example OTUs relative to total read counts obtained from sequencing of each fraction for the unlabelled (blue) and labelled (red) incubations for the different density fractions obtained. Panels B and E show the distribution of the absolute copy number of the example OTUs across the density gradient obtained from the data in (A) and (D) and the measured total copy number in each density fraction. Dashed lines depict calculated weighted average densities of the OTU used for qSIP calculations. Panels C and F show the distribution of copy numbers across the density gradient as the relative abundance of the maximum copy number (values between 0 and 1). Panels G and H reveal the stacked distributions (as shown in C and F) of all active OTUs in incubations with DOM (G) and protein (H). [Color figure can be viewed at wileyonlinelibrary.com]

**Fig. 4:**
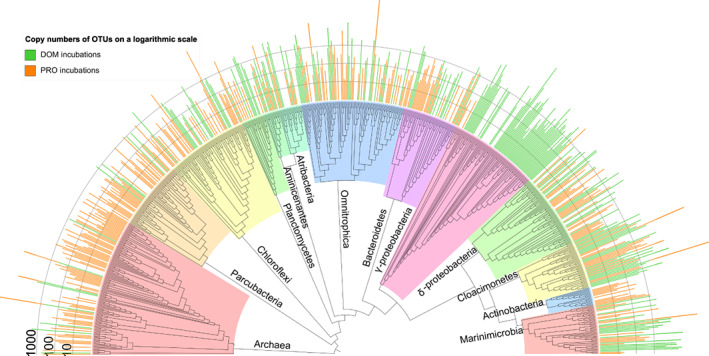
Taxonomic positioning of selected OTUs determined as active in the incubations with ^13^C/^15^N labelled dissolved organic matter (DOM, green bars) and ^13^C/^15^N labelled protein (PRO, orange bars). Colours on phylogenetic tree correspond to groupings at the Phylum level. Abundance bars are shown on a logarithmic scale. [Color figure can be viewed at wileyonlinelibrary.com]

A quantitative SIP (qSIP) approach (Hungate *et al*., 2015) was applied to determine the labelled—and hence active—organisms in the incubations. Briefly, with this method, the identity of labelled organisms was determined by comparing the weighted average density of the 16S rRNA gene from unlabelled to labelled incubations (Fig. 3B and D). A shift to a heavier density of a specific OTU is considered to be due to uptake of the label and, hence, activity. In addition, the GC content of the gene is evaluated based on the density in unlabelled incubations and this can be used to estimate the percentage of labelling compared to the theoretical maximum density if all atoms of the DNA molecule were labelled. The quantitative level of labelling was therefore evaluated by calculating the theoretical molecular weight excess (MWE; see experimental procedures section for full explanation of all calculations). During the incubations, a relatively small fraction of the OTUs became labelled. In incubations with labelled DOM and protein, 6.1% (210 OTUs) and 2.2% (357 OTUs) respectively became labelled (Table 1; Fig. 3). Additionally, absolute copy numbers were calculated by multiplying the relative abundance of each OTU with the total 16S rRNA copies as determined by quantitative PCR (qPCR) analysis (Fig. 2E). The weighted average density of the active community was 1.716 g ml^−1^ for the labelled DOM incubations and 1.721 g ml^−1^ for labelled protein incubations, while the corresponding mean values considering all OTUs were 1.710 and 1.708 g ml^−1^, showing a clear shift in the 16S rRNA gene density (Fig. 3; see Table S1 for all calculated qSIP values). Most of the labelled OTUs were only detected in incubations with one of the substrates; only 2% of the labelled OTUs were shared between labelled communities of the DOM and PRO incubations. However, the taxonomic positioning of the labelled OTUs was predominantly across the same phylogenetic groups in both incubation types and predominantly belonged to the *Marinimicrobia*, *Cloacimonetes*, *Chloroflexi*, *Deltaproteobacteria* and *Omnitrophica* (Fig. 4).

**Table 1:** The taxonomic affiliations and abundance values of the most common OTUs determined active in incubations with ^13^C/^15^N labelled DOM and ^13^C/^15^N labelled protein substrate.

	Phylum	Class	Labelled DOM incubations			Labelled protein incubations		
			Number of active OTUs	Total sequences	Active OTUs (%)	Number of active OTUs	Total sequences	Active OTUs (%)
*Bacteria*	*Actinobacteria*		0	0	0	11	2613	3.1
	*Chlorobi*		5	8386	2.4	17	3375	4.8
	*Chloroflexi*	*Anaerolineae*	1	5575	0.5	3	138	0.8
		*Dehalococcoidia*	15	9213	7.1	21	10 650	5.9
		*Thermoflexia*	0	0	0	6	2088	1.7
	*Cloacimonetes*	*MSBL2*	19	137 700	9.0	18	25 713	5.1
		*MSBL8*	5	18 312	2.4	3	3913	0.8
	*Marinimicrobia*		18	202 225	8.6	16	17 788	4.5
	*Omnitrophica*		24	55 513	11.4	43	14 925	12.1
	*Parcubacteria*		3	1950	1.4	28	6025	7.9
	*Proteobacteria*	*Alphaproteobacteria*	1	138	0.5	1	125	0.3
		*Deltaproteobacteria*	16	47,400	7.6	30	10 975	8.4
		*Epsilonproteobacteria*	1	2338	0.5	0	0	0
		*Gammaproteobacteria*	59	549 075	28.1	0	0	0
*Archaea*	*Woesearchaeota (DHVEG‐6)*		5	6088	2.4	73	11 175	20.5
		Total	210	1 × 10^6^	100	357	1.3 × 10^5^	100

For the DOM‐utilizing microorganisms, 16S rRNA gene sequences affiliated to the *Gammaproteobacteria* of the *Psychromonas* genus were dominant (28%) among the labelled OTUs, and a total of 5.4 × 10^5^ copies, with a single OTU with 3.8 × 10^5^ copies (Table 1). The next most abundant labelled OTUs were from *Omnitrophica* (11.4%), followed by the group SEEP‐SRB1 and *Desulfatigans* from the *Deltaproteobacteria* (both 3.8%), *Marinimicrobia* (8.6%) and the class MSBL2 of *Cloacimonetes* (11.4%). In contrast, the most abundant OTUs in terms of relative copy numbers after *Gammaproteobacteria* were those affiliated to *Marinimicrobia* (2 × 10^5^ copies) and to *Cloacimonetes* (1.4 × 10^5^ copies). There were only five OTUs with an MWE of >35%; three *Proteobacteria*, one *Omnitrophica* and one *Marinimicrobia*.

Regarding the protein‐utilizing microorganisms, *Woesearchaeota* was the most active phylum with a 20.5% relative abundance of the active community. However, its total abundance in copy numbers was relatively low (1 × 10^4^ copies, Table 1). The next most active taxa in terms of labelled OTUs were *Omnitrophica* (12.1%), followed by *Parcubacteria* (7.9%), the *Chloroflexi* class *Dehalococcoidia* (5.9%), *Cloacimonetes* MSBL2 (5.1%), the *Deltaproteobacterial* genera SEEP‐SRB1 (4.5%) and *Desulfatiglans* (3.6%), *Chlorobi* (4.8%) and *Marinimicrobia* (4.5%). The majority of relative copy numbers were from *Cloacimonetes* (3 × 10^4^ copies), followed by the *Marinimicrobia* (2 × 10^4^ copies) (Table 1). Organisms with an MWE of >35% (33 OTUs) belonged predominantly to the candidate phyla *Parcubacteria* and the DPANN archaeal phyla *Woesearchaeota*.

### Analysis of MAGs taxonomically related to the active community

We compared the active microbial community detected both in DOM and in protein‐amended incubations with the genomic potential for OM degradation of MAGs obtained from the Black Sea water column in 2013. We chose the analysed MAGs based on their quality, completeness and affiliation to the phyla that were determined as active in the DNA‐SIP incubations performed during the 2017 campaign. Phylogenetic trees showing the relationship of closely related genomes with 16S rRNA genes to the active OTUs can be found in Figs S3‐S8. We analysed metabolic genes of the selected MAGs using a selection of protein databases (i.e. KAAS and TIGRFAM; see section Experimental procedure for detailed methods). Additionally, we searched for genes encoding for carbohydrate‐active enzymes (CAZymes) and extracellular peptidases (MEROPS and SignalP or PRED‐SIGNAL) to estimate the substrate degradation potential of the MAGs. We analysed three MAGs affiliated to the bacterial phyla *Chloroflexi* (bin31, bin47 and bin52), two from *Proteobacteria* (bin40 and bin81 both belonging to the *Deltaproteobacteria*), and one each from *Marinimicrobia* (bin1), *Omnitrophica* (bin146) and *Cloacimonetes* (bin17). Two MAGs belonging to the archaeal phylum *Woesearchaeota* (bin111 and bin61) were also analysed (Table 2). The MAGs ranged from 56% to 96% in completeness with a contamination between 0% and 10% (Table 2). The *Woesearcheaota* MAGs as well the *Omnitrophica* MAG had a small estimated genome size of about 1.5 Mb, while the largest estimated genome sizes belonged to *Chloroflexi* bin30 (5.7 Mb) and the *Deltaproteobacteria* bin40 (4.6 Mb). The abundance of the co‐assembled reads belonging to each MAG at the 1000 m depth metagenome ranged from 8% (*Marinimicrobia* bin1) to 0.001% (*Woesearchaeota* bin111) (Table 2). Of the annotated proteins (*n* = 821–4286) a larger percentage of genes were assigned hypothetical for archaea (mean 58%) than for bacteria (mean 43%).

**Table 2:** Assembly statistics of MAGs acquired from the metagenomes obtained in 2013.

Bin No.	Phylum	Class	Family/Genus	Compl^a^ (%)	Cont^b^ (%)	Estimated genome size (Mbp)^c^	% reads mapped	No. of contigs	No. of annotated proteins	IMG accession
bin1	*Marinimicrobia*			89.0	1.0	2.24	8.005	232	1708	Pending
bin146	*Omnitrophica*			56.5	9.6	1.43	0.045	230	821	2814122937
bin17	*Cloacimonetes*			95.6	0.0	2.03	2.141	230	1612	2737471814
bin31	*Chloroflexi*			80.9	4.6	5.71	2.346	612	4132	2806311051
bin47	*Chloroflexi*	*Dehalococcoidia*		71.0	2.0	1.95	0.890	234	1355	2806311052
bin52	*Chloroflexi*	*Anaerolineales*		60.9	7.0	4.01	1.621	615	2500	2806311053
bin40	*Proteobacteria*	*Deltaproteobacteria*	*Desulfatiglans*	94.7	4.7	4.58	1.289	487	4286	2814122938
bin81	*Proteobacteria*	*Deltaproteobacteria*	*Desulfobulboceae*	96.4	0.0	3.13	0.007	227	2828	2814122939
bin111	*Woesearchaeota*			84.6	5.8	1.46	0.001	113	1440	2806311056
bin61	*Woesearchaeota*			86.6	0.5	1.46	0.054	39	1369	2806311055

The most detailed known taxonomic information is shown.

a. Compl: completeness.

b. Cont: contamination.

c. Genome size was estimated from the bin size corrected for the degree of contamination and degree of completeness; Mbp: Millions of base pairs.

### Central metabolic pathways of the MAGs

**Fig. 5:**
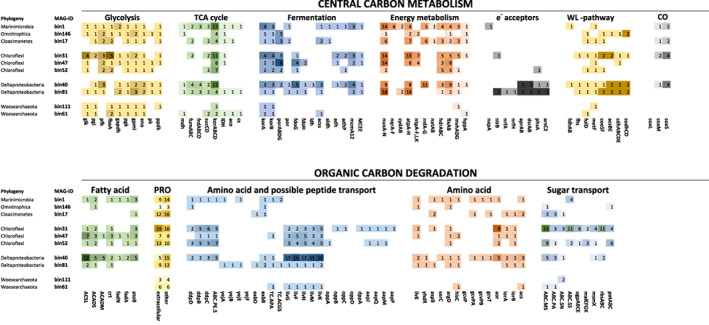
Comparison of selected genes involved in central carbon metabolism, energy metabolism and protein degradation between the MAGs discussed in this article. See full explanation of gene names and counts in Table S2. Darker colours correspond to higher counts in each category, while the lightest colour is always 1 copy of each gene. WL‐pathway: Wood‐Ljungdahl pathway of acetogenesis, CO: carbon monoxide processing genes, PRO: Proteases. [Color figure can be viewed at wileyonlinelibrary.com]

A range of 416–1645 genes were categorized into cell functions from the KEGG database (Fig. 5, Table S2). A near‐complete glycolysis pathway was found in all MAGs, as well as a diverse range of genes involved in fermentative metabolism, such as 2‐oxoglutarate/2‐oxoacid and pyruvate ferredoxin oxidoreductases (*korAB*, *porABDG*), formate dehydrogenases (*fdoGH*), methylmalonyl‐CoA mutase (*mcmA*) and alcohol dehydrogenases (*adh*).

Energy conservation genes involved in oxidative phosphorylation, such as NADH oxidoreductases (*nuoA‐N*) and ATP synthases (*atpA‐H/ntpA‐K*) were found in all MAGs, except in the *Woesearchaeota* MAGs (bin111, bin61). In addition, a Na^+^ transporting NADH‐quinone oxidoreductase (*nqrA‐F*) was found in bin1 (*Marinimicrobia*). Only a few genes suggesting the use of terminal electron acceptors were identified. Genes for nitrogen cycling (nitrite reductase *nirB*, *nrfAH*; nitrogenase *nifDHK*) were mainly found in bin81 (*Deltaproteobacteria)* and a periplasmic nitrate reductase (*napA*) was found in bin1 (*Marinimicrobia*). Dissimilatory sulphur cycling genes (sulfite reductase, *dsrAB*, polysulfide reductase *phsA*) were present in *Deltaproteobacteria* (bin40 and bin81). Potential arsenate reductase genes (*arsC2*) were found in the *Deltaproteobacteria* MAGs (bin40, bin81) and possible selenate reductase genes (*ygfKM*) in bin31 (*Chloroflexi*). Oxygen‐reducing ubiquinol oxidases (*cydAB*) were found in bin1 (*Marinimicrobia*) and bin81 (*Deltaproteobacteria*). Potential for using hydrogen with NiFe ‐hydrogenase (*hyaABD*) was found in bin47 (*Chloroflexi*) and bin40 (*Deltasulfatiglans*). Bin40 also contained a second NiFe ‐hydrogenase (hydAB).

Several complexes were found that drive alternative intracellular redox cycling through the electron carriers NADH and ferredoxin (Fd). An Rnf–complex was found in bin1, bin17 and bin40. Heterodisulfide reductase (*hdrABC*) was encoded in all bacterial MAGs except bin146 (*Omnitrophica*). In addition, the *mvhADG* gene complex was encoded in MAGs bin1, bin17 and bin31. The *fixAB* electron transfer flavoprotein was encoded in all MAGs except in the *Woesearchaeota* (bin111 and bin61). We also found the *Metf* methylenetetrahydrofolate reductase in most MAGs as well as the glycine cleavage system in bin1 and bin40. The full Wood‐Ljungdahl pathway was encoded in the MAGs of *Chloroflexi* bin31 and the *Deltaproteobacterial* MAGs bin40 and bin81.

### OM degradation potential of the MAGs

**Fig. 6:**
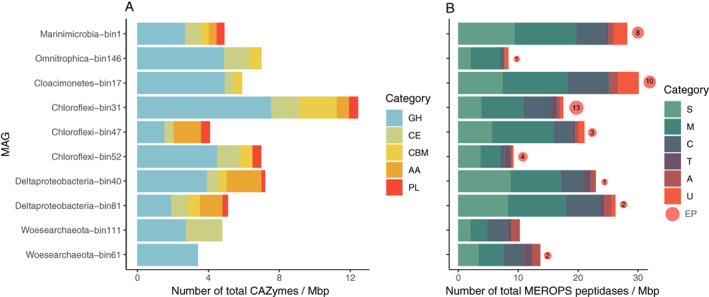
Number of genes encoding for substrate processing categories by MAG normalized to estimated genome size (Mbp: Millions of base pairs). A. Carbohydrate active enzymes, AA; auxiliary activities, CBM; carbohydrate‐binding modules, CE; carbohydrate esterases, GH; glucoside hydrolases, PL; polysaccharide lyases, and B. Peptidases identified by the MEROPS database and extracellular peptidases with Signal‐P or PRED‐SIGNAL, S; serine peptidases, M; metallopeptidases, C; cysteine peptidases, T; threonine peptidases, A; aspartic peptidases, U; unknown peptidases, and EP; extracellular proteases, amount shown with the size and total number inside the red circles (not normalized). [Color figure can be viewed at wileyonlinelibrary.com]

The CAZyme and MEROPS databases were used to evaluate the genomic potential for the degradation of different groups of organic compounds in the MAGs (Fig. 6A, Table S2). The most common carbohydrate esterases (CEs) detected belonged to the families 4 and 14. The first gene potentially encoding the esterase was detected three times in the *Woesearchaeota* bin111 and twice in the *Omnitrophica* bin146 and includes acetyl xylan esterases and chitin as well as peptidoglycan deacetylases. On the other hand, family 14 CEs acting on C─N bonds in linear amides were detected in the *Chloroflexi* bin31 and bin52. The most common carbohydrate‐binding module was from family 50, commonly found in enzymes cleaving chitin or peptidoglycan and mainly detected in the *Chloroflexi* bin31. The most abundant auxiliary activity was from family 4, which catalyses the conversion of a wide range of phenolic compounds and was mainly detected in the *Desulfatiglans* bin40. The quantity of identified CAZy was highest in one of the *Chloroflexi* MAGs (bin31, total assigned CAZy; *n* = 71) and the MAG classified as *Desulfatiglans* (bin40, *n* = 33, Fig. 6A).

The identified peptidases were diverse, with few overlaps between the analysed MAGs. Most peptidases (also extracellular) were found in the *Chloroflexi*, *Cloacimonetes* and *Marinimicrobia* MAGs (*n* = 13, 10 and 8 extracellular peptidases respectively, Fig. 6B). Commonly found extracellular peptidases were serine endopeptidases like subtilisin (S8A), chymotrypsin (S1C), and cysteine endopeptidases like gingipain (C25). Three different types of bacterial cell wall hydrolysing peptidases (C82A, M23A and M23B) were found in *Chloroflexi* bin 31.

In addition, we searched for substrate degradation pathways and related transporters (Fig. 5). Genes for fatty acid degradation through the beta‐oxidation pathway were more complete in the MAGs *Chloroflexi* bin47 (19 copies of seven different genes; Fig. 5) and *Desulfatiglans* bin40 (32 copies of seven different genes; Fig. 5). General capabilities for amino acid degradation through the use of aminotransferases to produce 2‐keto acids and ferredoxin oxidoreductases (*aor*, *iorAB*) to metabolize these to aldehydes or acetyl‐coA were found in most bins, with the exception of the *Woesearchaeota* MAGs (bin111 and bin61). Branched‐chain amino acid transporters were the most common ABC transporters (*livGHMK*) with up to 16 copies found in MAGs affiliated to the phyla *Chloroflexi* (bin31, bin47 and bin52) and *Deltaproteobacteria*, (bin40 and bin81). The ABC‐type peptide/nickel transporters (*ddpBCD*) were also identified in multiple copies in *Chloroflexi* (bin31), *Deltaproteobacteria* (bin31, bin52, bin40) and in single copies in *Marinimictobia* (bin1) and in *Dehalococcoidia* (bin47). In addition, the most complete *Chloroflexi* MAG (bin31) had multiple sugar transporters matching its diverse CAZyme repertoire.

## Discussion

The aim of this study was to identify the microbial community degrading labile OM in a marine euxinic water column and to determine if this community is defined by the available substrate type. With the combination of exploring the genetic potential using metagenomics and an incubation study to define active taxonomic groups, we are able to better understand the metabolic roles of the uncultivated majority of the diverse microbes in this environment. These complementary, cultivation‐independent approaches were applied in tandem to estimate how well metabolic predictions from genomic information match actual activity detected in the specific process of OM degradation.

The microbial community at 1000 m in the Black Sea was comprised of uncultivated microbial taxa ubiquitously occurring in anoxic environments like *Chloroflexi* (Hug *et al*., 2013a) and *Deltaproteobacteria* (Jochum *et al*., 2018; Skennerton *et al*., 2017), or specific to oceanic oxygen minimum zones worldwide, like *Marinimicrobia* (Hawley *et al*., 2017), as well as of microbial groups that remain poorly characterized like *Cloacimonetes* (Pelletier *et al*., 2008) and *Omnitrophica* (Rotaru *et al*., 2012). The composition of this community was similar in 2013 and 2017 (Fig. 2A), showing that the biogeochemical factors defining the microbial community do not vary much with time in the deep waters of the Black Sea, as would be expected from the strong stratification of the large basin. We compared the active OTUs in an incubation to the MAGs obtained from the same site that were affiliated to the same phyla. Though they are not directly linked, we consider the obtained MAGs to be the closest available genetic information to the studied OTUs, and to give valuable evidence on the genetic potential of the microbial community in the Black Sea.

### DOM‐utilizing microbes

Few phyla were found to be labelled in only one of the two provided substrate incubations. Moreover, these substrate‐specific groups made up a major part (10%–30%) of the active OTUs in each incubation type, indicating that their activity was directly linked to the compound type of the provided substrate. In the active organisms in DOM incubations *Gammaproteobacteria* genus *Psychromonas* was the most abundant taxa (Fig. 2B). These organisms have previously been connected to rapid sugar fermentation in RNA‐SIP incubations with tidal‐flat sediments (Graue *et al*., 2011). Additionally, members of the closely related genus in *Gammaproteobacteria* are well‐known copiotrophs and have been detected in bottle incubations with or without substrate additions (Nelson and Carlson, 2012, Stewart *et al*. 2012). This phenomenon is deemed the bottle effect and possibly has to do with physical changes induced by sampling strategies in marine research. However, the OM provided in our incubations is labile and represents fresh phytoplankton biomass, which is not expected to be abundant at 1000 m depth in the sulphidic water column. The total absence of the *Gammaproteobacteria* from the active protein‐degrading community (and minor contribution to the total community) gives an indication that in our incubations the bioavailability of the substrate and the dominance of labile molecules enabled the growth of a copiotrophic organism, not significantly present in the deeper reduced water column of the Black Sea. The labelling of this genus after only half a day in previous anoxic experiments supports this conclusion (Graue *et al*., 2011). In addition to this, the DOM incubations had eightfold the amount of 16S rRNA gene sequences as determined by qPCR (Fig. 2E) compared to the protein incubations, also indicating a more rapidly growing microbial community and therefore possibly more readily usable carbon sources in the DOM carbon pool.

### Protein‐utilizing organisms

The active groups that were specific to the protein‐amended incubations at the phylum level are from organisms with conspicuously small genomes; the DPANN archaea *Woesearchaeota*, and the bacterial phyla *Parcubacteria* (Nelson and Stegen, 2015; Liu *et al*., 2017; Castelle *et al*., 2018). In fact, the most active phylum, the *Woesearchaeota*, had about double the number of active OTUs than the next most abundant phylum. In addition, the most labelled organisms in terms of MWE were also predominantly affiliated to the *Woesearchaeota*. This high level of labelling would suggest that this organism could be utilizing the protein substrate for cellular growth, and therefore incorporating its carbon and nitrogen into its DNA. However, the MAGs classified as *Woesearchaeota* retrieved from the study site have relatively few genes assigned to CAZymes and few extracellular peptidases. This group belongs to the recently described DPANN superphylum of archaea, which is found worldwide in diverse habitats, with a preference for anoxic environments (Liu *et al*., 2018). Similar to previous studies on DPANN genomes, the two *Woesearchaeota* MAGs had considerably small genomes, seemingly lacking many essential metabolic genes, and in the case of bin111 also lacking atricarboxylic acid cycle and amino acid transporters, pointing to nutritional dependencies on other organisms (Castelle *et al*., 2015, 2018; Liu *et al*., 2018; Dombrowski *et al*., 2019). It is also possible that these genes were missed as the MAGs are not 100% complete. Conclusive genomic information to explain the success of this group is not easy to acquire as the taxonomic diversity and also metabolic versatility of the genomes acquired is large and a majority of the detected genes remain uncharacterized. Furthermore, more targeted studies will be required to understand the mechanism by which these organisms can incorporate the substrate into their cells.

While the *Woesearchaeota* were an abundant phylum in the environmental samples based on 16S rRNA gene amplicon sequencing, the *Parcubacteria* groups were not detected in significant amounts in natural Black Sea waters, and, hence, we did not obtain a *Parcubacteria*‐related MAG from metagenomes sequenced from the study site. This may highlight the disconnection between abundance and activity and shows the importance of activity measurements in characterizing microbial processes. In this case, the activity of this group could also be caused by a lability of the substrate that is not relevant in the deeper Black Sea similar to the most active phylum in the DOM incubations. The similarities between these two substrate‐specific phyla in the protein incubations can, however, indicate a metabolic strategy that was induced in our experiment. Like *Woesearchaeota*, the members of the *Parcubacteria* phylum, which is part of the candidate phyla radiation (CPR), are hypothesized to have a lifestyle that is dependent on other organisms for many essential metabolites (Kantor *et al*., 2013; Brown *et al*., 2015; Nelson and Stegen, 2015; Hug *et al*., 2016; Castelle *et al*., 2018). These groups had also relatively low copy numbers that can be the result of their slow growth, though it is also possible that it is an artefact of PCR bias, arising from the fact that these taxonomic groups have usually only one copy of the 16S rRNA gene in their genomes (Castelle *et al*., 2018). Our results indicate that the DPANN archaea and CPR bacteria, which seem to share a metabolic strategy (Castelle *et al*., 2015, 2018; Nelson and Stegen, 2015; Paul *et al*., 2017; Liu *et al*., 2018; Dombrowski *et al*., 2019), form an important part of the protein‐degrading microbial communities in the anoxic waters of the Black Sea. The role of archaea in degrading proteins especially in anoxic marine sediments has been hypothesized (Lloyd *et al*., 2013; Castelle *et al*., 2015). Orsi *et al*. (2016) reported that MGII Euryarchaeota were active in protein degradation in surface waters. On the other hand, protein was the only substrate out of several tested that was not assimilated by Crenarchaeota in salt marsh sediments (Seyler *et al*., 2014). The activity of DPANN Woesearchaeota in our incubations would, therefore, be confirming the role of an archaeal phylum in protein degradation in an anoxic environment for the first time.

Previous studies testing the microbial communities degrading proteins in marine environments found completely different microorganisms from those detected in our incubations (cf. Orsi *et al*., 2016 ; Liu *et al*., 2017). A diverse community was detected with abundant members of Gamma‐ and Alphaproteobacteria as well as members of the Flavobacteria and Verrucomicrobia in oxic waters (Orsi *et al*., 2016; Liu *et al*., 2017). The only similarity we find to the oxic incubations is a considerable activity of Actinobacteria in the assimilation of protein substrate (Table 1), as also detected by the studies of Orsi *et al*. (2016) and Liu *et al*. (2017). The Actinobacteria sequences in our incubations belonged almost completely (10 out of 11 OTUs) to the uncultured group OPB41, which surprisingly previously has been linked to the degradation of sugars in subsurface sediments (Bird *et al*., 2019). Our results could suggest a more widespread role for members of the Actinobacteria in protein degradation in the anoxic marine water columns. Liu *et al*. (2017) found a strong difference in the active microbial community degrading peptides when incubations with oxic waters were compared to that under hypoxic conditions but it was still very different from the one in our experiment. This difference may well be explained as the sulphidic waters incubated in our experiments have a much lower redox potential than the hypoxic waters incubated in their experiments. This would mean that, at least in the case of protein degradation, environmental conditions would be a more defining factor for the community than the substrate type provided. More comparison across the full water column and with more specific substrate sources would help answer these questions.

### Taxonomic groups active in both substrate incubations

In addition to the main degraders forming a major part of the active community discussed in the previous section, several uncultivated microbial groups that belong to the most abundant taxonomic groups in the original water column were found to be active at both substrate incubations. Members of the phyla *Omnitrophica*, *Marinimicrobia*, *Cloacimonetes*, *Chloroflexi* and the order *Deltaproteobacteria*, each comprised 5%–12% of the total active OTUs in each incubation experiment.

*Omnitrophica* spp. were the second most active in both incubations with 11%–12% of active OTUs. We detected only a few genes for polymer degradation in the *Omnitrophica* MAG (bin146; Fig. 6), making it unlikely that these organisms are capable of the initial degradation of the provided substrate (i.e. algal proteins or DOM). *Omnitrophica* spp. have been linked to a host‐attached lifestyle in methanogenic reactors (Rotaru *et al*., 2012), and therefore its success in both substrate incubations indicates a metabolic strategy not linked to a specific carbon source but possibly to an actively growing host population (Rotaru *et al*., 2012; Kizina* et al*., 2017). The *Omnitrophica* MAG acquired from the Black Sea was the least complete of our analysed genomes (56%), making it difficult to infer the presence of full degradation pathways. However, we find partial pathways for OM degradation through glycolysis to acetate as well as evidence for hydrogen production with formate hydrogenlyase, and electron transfer with *fixAB*. Genes related to hydrogen production in this group has previously been detected from hydrothermal sediments (Dombrowski *et al.,* 2017). Similar to Kizina *et al*. (2017), we find genes for the Type II secretion/Type IV pilus assembly system possibly linked to attachment to a host cell. Our *Omnitrophica* MAG could possibly perform hydrogen and acetate producing fermentation by scavenging low‐molecular‐weight OM from its environment.

The *Marinimicrobia* and *Cloacimonetes* phyla were the next most abundant shared organism groups in our DNA‐SIP incubations. The MAGs affiliated to these groups contained the genes encoding complete central metabolic pathways like glycolysis and enzymes for the degradation of amino acids, with several extracellular peptidases and the largest collection of energy metabolism genes of the assessed MAGs (Figs 5 and 6). They encode the electron transferring complexes and hydrogenases (*Rnf*, *MvhHdr* and *FixAB*) known from highly reduced conditions in methanogenic reactors (Nobu *et al*., 2015). The *Rnf* complex is capable of either coupling the reduction of NAD^+^ to the oxidation of reduced Fd and translocation of a proton across the membrane or the reverse, and is sometimes found in connection with the electron‐transfer‐flavoprotein‐oxidizing hydrogenase *FixAB* (Nobu *et al*., 2015). On the other hand, *MvhHdr* is an electron bifurcating hydrogenase, which couples the reduction of ferredoxin and a heterodisulfide from CoM‐CoB to the oxidation of hydrogen. This enables an organism to drive a thermodynamically unfavourable endergonic reaction with the reduction of two electron acceptors (Poudel *et al*., 2018). While *Marinimicrobia* spp. have been described with diverse capabilities to use alternative electron acceptors (Wright *et al*., 2014; Hawley *et al*., 2017; Plominsky *et al*., 2018), recently obtained MAGs from oxygen minimum zones and the hypoxic northern Gulf of Mexico suggested also a niche for degradation of macromolecular OM and peptides (Bertagnolli *et al*., 2017; Thrash *et al*., 2017). On the other hand, the elusive *Cloacimonetes* have been mainly linked to amino acid (Pelletier *et al*., 2008), cellulose (Limam *et al*., 2014) and propionate degradation (Nobu *et al*., 2015; Dyksma and Gallert, 2019). The *Cloacimonetes* MAG encodes pathways for the degradation of several amino acids but only one gene of those necessary for propionate degradation (*pccB*) was identified on a short contig (Fig. S11). Despite the considerable amount of extracellular peptidases encoded by the MAGs belonging to both phyla (Fig. 6), our DNA‐SIP study did not indicate a specific preference for the proteinaceous substrate. Instead, the *Marinimicrobia* and *Cloacimonetes* spp. could act as initial degraders of high‐molecular‐weight OM (including protein), and therefore have a role as generalist fermentative heterotrophs, ensuring their survival and persistence with many carbon sources. Their success could be based on being able to use complex carbon substrates while using diverse strategies to discard electrons in a highly reduced environment.

The *Deltaproteobacteria* and (*Chloroflexi*) *Dehalococcoidia* spp. also occurred in similar abundances in the DNA‐SIP incubations with the different substrates. The obtained MAGs affiliated to these phyla suggest capabilities for the degradation of intermediate products in an interaction with primary degraders. The *Deltaproteobacterial* phylum was comprised of two main known sulphate‐reducing genera, *Desulfatiglans* from the *Desulfurellales* class (Jochum *et al*., 2018), and the SEEP‐SRB1 group from the *Desulfobacterales* (Skennerton *et al*., 2017). The MAG affiliated to *Desulfatiglans* (bin40) contained, in addition to *dsrAB*, 10 copies of branched‐chain amino acid ABC transporter genes, and genes encoding formate dehydrogenases, a full pathway for fatty acid degradation and the enzymes required for the utilization of propanoate and butanoate, all important indicators for the degradation of intermediate carbon compounds resulting from primary fermentation (Schink and Stams, 2013; Narihiro *et al*., 2016). The other detected *Deltaproteobacteria* MAG (bin81) also contained a *dsrAB*, genes of the propanoate conversion pathway, as well as the branched‐chain amino acid transporters and genes for the degradation pathways similar to those of the *Desulfatiglans* MAG. *Dehalococcoidia*, on the other hand, have been defined by their capabilities for dehalogenation as a terminal reduction pathway, though recently genomes retrieved from the deep biosphere have classified these bacteria as anaerobic acetogens capable of fermentation of plant polymers as well as amino acids and organosulphur compounds (Hug *et al*., 2013a; Wasmund *et al*., 2014). In the *Dehaloccoidia* MAG (bin47), we did not find indications of complex substrate degradation, the Wood‐Ljungdahl pathway, or a reductive dehalogenase gene, but several copies of genes for the beta‐oxidation of fatty acids (*fadD*, *fabG*), for formate (*fdoGH*) and hydrogen utilization (*hdrABC*, *fixAB*), and propionate degradation (*mcmA1/2*, *MCEE*), connecting it to a similar metabolic role in the community as the *Deltaproteobacteria*. Based on this metabolic analysis, the MAGs belonging to these phyla suggest capabilities for the degradation of intermediate fermentation products in close interaction with other organisms. The *Deltaproteobacteria* most likely use sulfuric compounds as electron sinks, while the possible electron acceptor used by *Dehalococcoidia* is not evident from the MAG analysis. Based on the ubiquity of dehalogenation in this taxonomic group, could be utilizing organohalide compounds (Hug *et al*., 2013b; Biderre‐Petit *et al*., 2016). In contrast with the *Dehalococcoidia*, other taxonomic groups belonging to the phylum *Chloroflexi* were low in abundance in the active community as evident from the qSIP experiments. The other *Chloroflexi* MAGs (bin31 and bin52) contained by far the most CAZy and extracellular peptidases of all evaluated MAGs. Therefore, the lack of labelling with the provided substrates is surprising and attests to a different metabolic strategy that possibly does not benefit from the addition of the labile carbon sources supplied. In addition, in a previous SIP study, members of the phyla Chloroflexi were active in both heterotrophic and autotrophic incubations with freshwater sediments (Coskun *et al*., 2018), so it is possible that these organisms would have an autotrophic or mixotrophic strategy for carbon assimilation. This confirms the importance of activity measurements in addition to the analysis of the metabolic potential of a microbial community.

Many of these phyla that were active in both substrate incubations belonged to the most common taxonomic groups from the original water column microbial community. These organisms seem to be able to persist though not proliferate in incubations with both protein and DOM. This could be an indication of a metabolic strategy based on the potential to utilise diverse complex carbon sources or intermediates of the degradation process to sustain cellular activity. The labelling of organisms in both substrate types that are possibly responsible for the degradation of intermediate fermentation products supports the findings from the genetic analysis, as these secondary fermenters would presumably be less reliant on the composition of the original substrate.

## Conclusions

Our study has revealed that the euxinic water column of the Black Sea harbours a microbial community highly adapted to the utilization of diverse electron cycling metabolisms while oxidizing the organic carbon compounds available in their environment. Many genomic features common to those reported for methanogenic reactors (Nobu *et al*., 2015) and deep subsurface environments (Poudel *et al*., 2018) are found in MAGs from our study, showing that utilisation of low redox potentials is one of the main defining features of life in this euxinic water column. In combination with the genomic information, our DNA‐SIP experiment indicates that members of the *Marinimicrobia* and *Cloacimonetes* phyla are generalists capable of utilizing macromolecular OM of different forms, while possibly relying on close interactions with other organisms to process fermentation products. According to our results, the degraders of these fermentation products could be organisms affiliated to *Deltaproteobacteria* as well as *Dehalococcoidia* from the phylum *Chloroflexi*. However, none of these organisms became dominant with the added labile carbon pools, suggesting that, as could be expected, the OM available in the Black Sea sulphidic zone at 1000 m is more diverse and/or recalcitrant than the OM provided in our incubations. In addition, the activity of cells affiliated to *Woesearcheota*, *Parcubacteria* and *Omnitrophica*, especially in the protein‐amended incubations, is an interesting phenomenon, which shows for the first time the potential competitive advantage these organisms with small genomes and lacking many essential metabolic pathways might have in a natural microbial community. The exact reason why these organisms are able to become the most active members in our incubations remains to be answered, but it likely requires that their exploitation of the metabolism of other organisms or leaking metabolites is efficient. Nonetheless, a streamlined metabolism appears to be an important part of the degradation of OM in low‐energy euxinic conditions. It is possible that the role of competition is less important as a shaping force of the community than environmental pressures; something that has been suggested for oxygen‐depleted water columns previously (Bryant *et al*., 2012). When competition for limiting nutrients or substrate is not the defining factor of the community, but rather the limitations exposed by energetic constraints and possibly the sulphide concentration, it is more likely that redundant metabolic strategies can coexist within organisms that can cope with the environmental constraints. This could explain why organisms that are phylogenetically distant express the same metabolic strategy. Future incubations with pure amino acids or isotopic confirmation with microscopic techniques would give more insight into their ecological strategy and potential interactions with other organisms.

Finally, our study shows the importance of estimating the activity of microbial communities to enhance our understanding of the meaning of genetic adaptations to specific environmental conditions. Solely genomic data can give a good overview of metabolisms available in the community but to understand the defining dynamics of natural microbial communities more studies are required that also consider the consequences of these genomic adaptations. Therefore, the use of novel methods to follow activity in microbial communities is paramount to broaden our understanding of natural phenomena and metabolic versatility, as well as the evolutionary pressures that affect life on earth.

## Experimental procedures

### Sample collection

Water samples were collected from the western basin of the Black Sea in Spring 2017 during R/V Pelagia cruise number 64PE418 from station 2 (42° 53.8′ N 30° 40.7′ E). Two hundred litres of water from 1000 m depth was collected with an ultra‐clean conductivity‐temperature‐density (CTD) system, which was equipped with among others a SBE3plus thermometer, SBE4 conductivity sensor and SBE43 dissolved oxygen sensor (Sea‐Bird Electronics, Bellevue, WA). Water column samples for nutrient analyses were collected from the same CTD bottles and preserved on board for chemical analyses. About 5 ml of the sample was filtered through a 0.2 μm Acrodisc syringe filter with a Supor membrane (Pall Corporation, New York, NY). Samples for NO_3_^−^, NO_2_^−^ and NH_4_ were frozen at −20°C in a pre‐rinsed pony vial. Samples for DIC were preserved at +4°C, and samples for HS were fixed with 40 μl 1 N NaOH, and frozen at −20°C. About 20 ml of water was collected and filtered for DOC and TN measurements into precombusted glass vials. Water column samples for DNA analysis were collected by filtration of approximately 6 L of suspended particulate matter directly from sample bottles through Sterivex 0.22 μm membrane filters (Merck, Burlington, MA) and immediately frozen in −80°C after collection. Water for incubations was collected from the bottom of the 27 L sample bottles, while purging with nitrogen, therefore minimizing contact with ambient atmosphere. Incubations were carried through in 50 L round PP carboys (216‐1738, VWR, Radnor, PA). Carboys used for the incubation experiments were acid‐washed, rinsed once with site‐water and purged for about 10 min with nitrogen with 10 cycles of overpressurising before collecting the incubation water. Over time oxygen will leach through the plastic material used, but considering the short time periods of incubation, the large volume of water and high sulphide concentrations, the oxygen available for organisms should be minimal.

Additionally, samples for metagenomics analyses were collected on the R/V Pelagia 64PE371 in June–July 2013. Sampling methods, chemical and microbiological analyses of the water column during this cruise have been previously reported by Sollai *et al*. (2018). The main difference to the sampling campaign in 2017 was that samples were collected with a 0.7 μm pore size glass fibre filter (GF/F).

### Substrate preparation

Two separate substrates were prepared from cultures of the diatom *Thalassiosira pseudonana* CYY9928. The diatom culture was grown in normal MDV media (Javaheri *et al*., 2015, Table S5) to generate unlabelled biomass, as well as grown in the same media supplemented with 30% ^13^C‐NaHCO_3_ (Cambridge Isotope Laboratories, Andover, MA), and 15% ^15^N‐NaNO_3_ (Sigma Aldrich, St. Louis, MO) to generate ^13^C/^15^N‐labelled biomass, for 4 weeks before biomass was collected by centrifugation. Pellets were washed three times with artificial seawater to remove residual labelled substrates, and freeze‐dried. An extract of DOM was prepared by adding 10 ml MilliQ water, 50 mg Devarda's alloy and 25 mg MgO for each 0.2 g freeze‐dried biomass to burst the freeze‐dried cells and eliminate any remaining dissolved inorganic nitrogen. The mixture was shaken for 48 h at room temperature (RT) and the supernatant was collected by centrifugation. The supernatant was subsequently filtered through a pre‐combusted 47 mm glass fibre filter (Whatman, Little Chalfont, UK), and a 0.2 μm syringe filter (Minisart®, Sartorius, Gottingen, Germany), before being freeze‐dried again and used as DOM substrate. Protein was extracted from the same collected biomass with a Trizol‐based method (Kirkland *et al*., 2006), and subsequently freeze‐dried to be used as the protein substrate. Protein amounts in each substrate were measured by the Bradford dye‐binding method with the Bio‐Rad protein assay kit (Bio‐Rad Laboratories, Hercules, CA). DNA contamination in the protein extracts was checked fluorometrically with Qubit™ dsDNA HS assay kit (Thermo Fisher Scientific, Waltham, MA). About 1 ng/μl DNA was remaining amounting to 0.02% w/w of the extract; this minor amount was considered to have minor effects on the substrate‐utilizing community. The remainder of protein extract weight is assumed to be mainly salts brought through from the extraction protocol. The atom‐% of label in the substrate was measured by EA‐IRMS (Flash 2000 EA with a DeltaV Advantage; Thermo Fisher Scientific, Bremen, Germany) equipped with a NC/NCS 3 m column, indicating a labelling of 11% ^13^C of the DOM, and 16% ^13^C of the measured protein in the protein extract (12% w/w).

### Incubation conditions

The substrate was added up to 2 h after the collection of the water. 67 mg of either freeze‐dried unlabelled DOM or ^13^C/^15^N DOM were dissolved in 10 ml of sterile‐filtered site seawater. Sixty‐seven milligrams of the freeze‐dried unlabelled protein extract and ^13^C/^15^N protein extract were dissolved into 15 ml sterile‐filtered site water, with 1% sodium dodecyl sulphate (SDS) and repeated heating in near‐boiling water. SDS denatures and dissolves proteins in water, changing the charge and exposing amino acid chains that would otherwise be hidden. The final concentration of SDS was diluted with an addition of about 3000× and, therefore, its effect on the incubations is considered minimal. Each of the substrates was dissolved in an ambient atmosphere and added through syringes to the 50 L incubation bottles while purging with nitrogen. The final concentration of the added substrate was 1.34 mg L^−1^, which is equivalent to the amount of DOC measured at these depths in the Black Sea (Ducklow *et al*., 2007). The bottles were thoroughly mixed and the headspaces were purged with nitrogen. The carboys were incubated on their side to minimize any gas leakage and shaken carefully after 1 day. The container was set to the *in situ* temperature of 8°C and kept in the dark. After 72 h of incubation, water was filtered on to 142 mm 0.2 μm polycarbonate filters (Isopore™, Merck, Burlington, MA) with a 0.45 μm nitrocellulose support filter (MF membrane, Sigma‐Aldrich, St. Louis, MO). The connecting tubing was acid‐washed in the beginning and rinsed thoroughly with milliq water between each treatment. The filters were collected and immediately frozen at −80°C until further processing back in the lab.

### DNA extractions

Water column samples collected in Sterivex filters were extracted with the Powersoil DNA Isolation kit (MoBio, QIAGEN, Carlsbad, CA, USA), according to the manufacturer's instructions but with an elution step with 50 μl TE. In addition, DNA was extracted from the 142 mm 0.2 μm polycarbonate incubation filters with a phenol/chloroform method (Sambrook and Russell, 2006). Briefly, filters were cut into small pieces with a sterile scalpel, and bead‐beaten with a vortex (VWR VV3, Radnor, PA) equipped with a custom adapter for 50 ml falcon tubes for 10 min at maximum speed with 1 g 0.1 mm zirconia beads (Biospec, Bartlesville, OK) and 15 ml of TE/sucrose buffer (6.7% sucrose, pH 8.0, 4°C). Supernatant was recovered, incubated at 37°C for 30 min with 0.8 mg ml^−1^ lysozyme (Thermo Scientific™, Waltham, MA), and further incubated at 65°C for 15 min 0.2 mg ml^−1^ Proteinase K (Invitrogen™, Carlsbad, CA) and 0.5% SDS. The mixture was cooled to 37°C and incubated again at 37°C for 30 min with 0.08 mg ml^−1^ RNase A. DNA was further extracted with one volume of phenol:chloroform:isoamyl alcohol (25:24:1), washed with chloroform:isoamyl alcohol (24:1) and chloroform. DNA was precipitated with 50 μg ml^−1^ glycogen, 0.3 M sodium acetate (pH 5.5), and 2.5 volumes of cold 70% ethanol. The pellet was dried and redissolved in PCR‐grade water. DNA concentrations were measured fluorometrically with Qubit™ dsDNA HS assay kit (Thermo Fisher Scientific), and extracts were stored at −80°C.

### Density gradient centrifugation and gradient fractionation

A density gradient centrifugation was done with CsCl gradients following the protocol of Dunford and Neufeld (2010). Briefly, 4 μg of DNA from each sample was added to a CsCl solution to the final density of 1.725 g ml^−1^ in 5.1 ml QuickSeal Polyallomer tubes (Beckman Coulter, Brea, CA). The samples were centrifuged for 60 h at 44 000 rpm (177 000 *g*_av_) at 20°C using a Vti 65.2 rotor (Beckman Coulter). The samples were then fractionated to 13 equal fractions, and the gradient formation was checked by measuring the density of each fraction from 10 μl of sample with a digital refractometer (AR2000 Reichert Technologies, Buffalo, NY). DNA was precipitated by adding 2 volumes of PEG solution (30% PEG6000, 1.6 M NaCl) and 20 μg of polyacrylamide as a carrier, incubating at RT for 2 h and pelleting the DNA by centrifugation at 13 000 ×rcf for 30 min at 4°C. Pellets were washed with 70% ethanol, let dry and resuspended in PCR‐grade water.

### 16S rRNA gene amplicon sequencing and analysis

The V4 region of the 16S SSU rRNA gene was amplified from DNA extracted both from the water column and from all fractions collected in the density gradient fractionation of the DNA‐SIP experiment (see above) with the forward primer 515F‐Y and the reverse primer 806RB (Caporaso *et al*., 2011; Apprill *et al*., 2015; Parada *et al*., 2016). PCR reactions were made in Phusion HF buffer with 0.2 mM dNTPs, 800 μg ml^−1^ BSA, 0.6 μM primers, 1 unit Phusion High‐Fidelity Taq polymerase (Thermo Fisher Scientific, Waltham, MA) and 5 μl of DNA template in a total of 50 μl. The conditions were 98°C for 30 s, 25 rounds of 98°C for 10 s, 50°C for 20 s, 72°C for 30 s, and finally, 7 min of extension at 72°C. All reactions were performed in triplicates and negative controls were added to each round. PCR products were run on 1% agarose gels for 50 min at 80 V with SmartLadder small fragment (100–1000 bp, Kaneka Eurogentec S.A., Seraing, Belgium) as a size marker. Target bands were cut out of the gel, and DNA was purified with the QIAquick Gel Extraction Kit (QIAGEN, Hilden, Germany). Samples were combined at equal concentrations, concentrated with the MinElute PCR Purification Kit (QIAGEN, Hilden, Germany) and sequenced at the University of Utrecht (Netherlands) on an Illumina Miseq platform as paired‐end reads of 250 bp.

Illumina amplicon data were analysed with an in‐house pipeline. Briefly, sequence quality was checked at several process points with FastQC (v. 0.11.3, Andrews, 2010). Paired‐end reads were extended with PEAR (v. 0.9.8, Zhang *et al*., 2013) with a minimum length of reads of 20, minimum overlap set at 7 and a *p*‐value threshold of 0.05. Barcodes were removed (rules: extract_barcodes –bc1_len 12 and correct_barcodes –bc_missmatch 2) and libraries were split (rule: split_libraries, max unacceptable phred –q 10, and maximum number of consecutive low base calls –r 5) and reads outside of 250–350 bp length were discarded (rule: remove_short_long_reads) with QIIME (v. 1.9.1, Caporaso *et al*., 2010). OTUs were picked with uclust with a 97% threshold and the most common sequence of each OTU cluster was picked as representative. The taxonomy of the representative sequences were assigned using uclust (75% cut‐off) against the SILVA database release 128 (Quast *et al*., 2012). As many sequences do not have more detailed taxonomic classification in the database, a cut‐off of 75% for phylum level taxonomy was used (Yarza *et al*., 2014). The taxonomic positioning of the sequences was confirmed with a phylogenetic analysis that included the alignment and trimming of sequences in ARB (v. 6.0.2, Westram *et al*., 2011) against the SILVA database release 128 (Quast *et al*., 2012). Sequences and selected neighbours of each taxonomic group were further aligned with MAFFT (v. 7.310, Katoh and Standley, 2013) and trimmed with TRIMAL (v. 1.2rev59, Capella‐Gutierrez *et al*., 2009) with the automatic method selection. A phylogenetic tree was then built from trimmed sequences with IQ‐TREE using automatic model selection and ultrafast bootstrap approximation (v. 1.6.7, Nguyen *et al*., 2015, Kalyaanamoorthy *et al*., 2017, Hoang *et al*., 2017) and visualized with iTOL (Letunic and Bork, 2016) (Supplementary Figs 3–8). 16S rRNA genes longer than 400 bp were collected from the metagenomes with barrnap (v. 0.9, https://github.com/tseemann/barrnap) with an e‐value cut‐off of 1e ^−20^. The taxonomy of these genes was analysed the same way as the amplicon data. The number of reads mapping to a gene was normalized by the total length of the gene and further to a relative abundance of all normalized reads Fig. 2A. All further analyses were carried out in the R package Phyloseq (v. 1.22.3, McMurdie and Holmes, 2013). For water column amplicon data sets, singletons were removed. For the substrate incubations, OTUs that had less than 13 sequences in each incubation (13 fractions per substrate) were removed prior to analysis as well as OTUs belonging to *Cyanobacteria*, as these could not be separated from remaining DNA contaminants from substrate addition. Finally, the remaining OTUs were normalized to the total read count per sample to obtain the relative abundance. The high numbers of OTUs we identified in our data sets may have to do with the clustering method (UCLUST) which has been known to result in overinflating OTU numbers when compared with mock communities (Edgar, 2017). Therefore, we will discuss the differences between treatments mainly in terms of relative abundances and do not consider an OTU as directly representing a microbial species. The 16S rRNA gene amplicon reads (raw data) have been deposited in the NCBI Sequence Read Archive under the BioProject number PRJNA596220.

### qPCR and qSIP analysis

For all fractions collected in the density gradient fractionation of the DNA‐SIP experiment where the sample was remaining, 16S rRNA gene copy numbers were estimated by performing a qPCR. qPCR was performed on a Biorad CFX96™ Real‐Time System/C1000™ Thermal cycler equipped with CFX Manager™ Software. qPCR reactions of a final volume of 25 μl were made in Phusion HF buffer, 0.2 mM, 0.6 μM of each of the primers used for the 16S rRNA gene amplicon sequencing analysis, 0.8 mg ml^−1^ of BSA, 0.2× SYBRGreen, 0.5 units of HF Phusion Taq polymerase and 4 μl of 10× diluted DNA template. All reactions were performed in triplicate and threshold values were compared to a calibration curve with concentrations ranging from 4.06 × 10^7^ to 4.06 × 10^2^ gene copies. Non‐template controls were included in every run. The PCR reactions were carried out with an initial denaturation step of 98°C for 30 s followed by 45 cycles of denaturation at 98°C for 10 s, annealing at 52°C for 20 s with plate read and elongation at 72°C for 30 s and a final elongation step at 80°C for 25 s. The specificity of the reactions was checked by a melting curve analysis. Copy numbers used for qSIP calculations are 16S rRNA gene copies found in total in each fraction of the density gradient. The abundance of prokaryotes (16S rRNA gene copy numbers l^−1^) were estimated by using the total copy numbers of all fractions in one sample normalized with the amount of DNA extracted from each incubation to estimate the seawater that was used to collect 4 μg of DNA used for each centrifugation. These are estimates, and should not be considered absolute, as it is probable that most error in this case comes from the amount of DNA originally extracted from the incubation filters.

We chose the qSIP analysis method (Hungate *et al*., 2015) to evaluate the incorporation of labelled substrate into the DNA, and consequently to identify ‘active’ OTUs with labelled ^13^C/^15^N DNA. Calculations were done using the QSIP function of the HTSSIP R package (Youngblut *et al*. 2018) with some modifications to also take into account ^15^N‐nitrogen assimilation. When calculating maximum heavy DNA molecular weight, we added the effect of nitrogen labelling to the functions in the R package. Since the nucleotides A and G have five nitrogen atoms, a maximum of 4.985 g mol^−1^ was added if fully labelled. For T with two nitrogen atoms the addition would be 1.994 g mol^−1^ and for C with three nitrogen atoms 2.991 g mol^−1^. Therefore, we modified equation (9) from Hungate *et al*. (2015) to account for nitrogen:

$$M_{\text{HEAVYMAXi}}=0.0025G_{i}+13.416+M_{\text{LIGHTi}}$$

The final calculation is thus not atom fraction excess (AFE) but MWE. In addition, we did not take into account the effect of natural isotope fractions in the calculation of MWE, as these have a minimal effect and in principal are already included in the calculations for unlabelled controls (*W*_light_ and GC content). Bootstrapping was not performed as we only had one replicate ^13^C/^15^N‐labelled and unlabelled control of each substrate. Because of the lack of statistical tests, we chose a stringent cut‐off value of the estimated MWE of 0.14, to assign OTUs as active. This matches approximately the proportion of label in the added substrate and, moreover, is considerably higher than the values Hungate *et al*. (2015) and Coskun *et al*. (2018) reported as average AFE of OTUs assigned as active in their experimental data. For more discussion on considerations for the use of this method, see Supplementary File 1, section 2. Phylogenetic analysis of the active OTUs was done with selected closest neighbours retrieved with the SILVA ACT tool. Alignment, trimming and tree‐building were done as previously described. Supplementary Fig. S2 shows the total workflow from incubations to determine active community members.

### Metagenomic and phylogenetic analysis of the MAGs

Sample collection, DNA extraction and metagenome sequencing and sequence analysis of water column samples collected from the Black Sea water column in a previous campaign in 2013 (64PE371, see above) is described in Villanueva *et al*. (2018). For some of the MAGs discussed here, 16S rRNA genes were identified with the CheckM ‘ssu_finder’ utility (v. 1.0.12; Parks *et al*., 2014). For the rest of the MAGs, Phylosift (v. 1.0.1; Darling *et al*., 2014) was used to extract marker genes for their phylogenetic placement as described in Dombrowski *et al*. (2018). The MAGs were then compared to all publicly available genomes in the NCBI database from the same taxonomic group, and the closest available 16S rRNA gene from a related MAG was added to the phylogenetic tree with the 16S rRNA genes of active OTUs to compare to the phylogeny of the active community (Figs S3‐S8). To determine the relative abundance, we mapped the scaffolds from each MAG against the metagenomics reads from the 1000 m water column sample using BBmap (v. 34.x, BBtools, Bushnell, B., sourceforge.net/projects/bbmap/). The percentage of reads mapped to each MAG was considered as an indicator of the abundance of the specific MAG at 1000 m depth. Genes were called using prodigal (v. 1.14‐dev, Seemann, 2014), and gene annotations performed against several databases. These include KAAS (KEGG automatic annotation server, v. 2.1; Moriya *et al*., 2007), TIGRFAM (v. 15.0; Haft *et al*., 2001), Pfam (v. 31.0; El‐Gebali *et al*., 2018 and Engel *et al*., 2017), CAZymes (v. 2; Lombard *et al*., 2014), Merops (version of November 2018; Rawlings *et al*., 2018), COG (version of November 2018; Tatusova *et al*., 2000) or arCOG (Makarova *et al*., 2015) and ncbi_nr (version of October 2018; O'Leary *et al*., 2016) databases with an e‐score threshold of 1e^−20^ for all except ncbi_nr (1e^−5^), MEROPS (1e^−80^), Pfam (1e^−10^) and COG (1e^−10^) databases. Annotations were performed using Hmmsearch (v. 3.1b2; Potter *et al*., 2018) and Blastp (v. 2.7.1; Altschul *et al*., 1997) or Diamond (v. 0.9.22; Buchfink *et al*., 2015). MAG comparisons were done with the identified KO hits for major carbon degradation and central metabolic genes (Dombrowski *et al*., 2018; Table S2), and an estimation of full pathways in each MAG separately was done by comparing the hits of all databases as well as localization in contigs. Extracellular peptidases were determined for MEROPS peptidase hits of MAGs using SignalP (Petersen *et al*., 2011) for bacterial MAGs and PRED‐SIGNAL (Bagos *et al*., 2009) for archaeal MAGs and manually curated by comparing to other database hits. All annotations can be found in Table S3. The MAGs described and discussed here are deposited in the Joint Genome Institute's integrated microbial genomes database (Table 2).

## Supporting information

**Supplementary file 1 Section 1**, A description of the main metabolic potential found in each evaluated MAG
**Section 2**, Considerations for DNA‐SIP
**Supplementary Fig. 1.** Above: Measured chemical parameters from the water column in μM; DOC: dissolved organic carbon, DIC: dissolved inorganic carbon, TN: total nitrogen. Below: CTD profiles across the water column.
**Supplementary Fig. 2.** Diagram of workflow from incubations to DNA‐SIP experiments
**Supplementary Figs 3–8.** Phylogenetic tree showing the 16S rRNA of active OTUs by phyla and a closest neighbouring 16S rRNA gene obtained from a publicly available genome closely related to the MAG of that phyla (based on 34 single‐copy marker genes) is shown.
**Supplementary Table 5.** List of 34 single‐copy marker genes used for phylogenetic analysis of MAGs
**Supplementary Fig. 9.** Chemical measurements from incubations at 72 h
**Supplementary Table 6.** Recipe for MDV media used for culturing the diatom biomass
**Supplementary Figs 10–13.** Reconstructed metabolic pathways of selected MAGs

**Supplementary file 2 Supplementary Table 1.** DNA‐SIP calculated parameters for all active OTUs in DOM incubations (Sheet 1), and PRO incubations (Sheet 2)
**Supplementary Table 2.** DNA‐SIP calculated parameters for all active OTUs in PRO incubations
**Supplementary Table 3.** List of KO annotations used for comparisons in Fig. 5.
**Supplementary Table 4.** Final list of extracellular peptidases bacteria MAGs
**Supplementary Table 5.** Final list of extracellular peptidases archaea MAGs
**Supplementary Table 6.** List of annotated CAZymes
**Supplementary Table 7.** List of annotated MEROPS peptidases
**Supplementary Table 8.** List of genes used for Supplementary Fig. 10

**Supplementary file 3 Supplementary Table 9.** All annotated genes in order by contigs for bacterial MAGs
**Supplementary Table 10.** All annotated genes in order by contigs for archaeal MAGs
