# Supplementary material for: A diverse uncultivated microbial community is responsible for organic matter degradation in the Black Sea sulphidic zone
Source: Environ Microbiol. 2020 Jan 13;23(6):2709–28. doi: 10.1111/1462-2920.14902 (PMC8359207; doi:10.1111/1462-2920.14902)
Supplement: Supplementary file 1 — Section 1, A description of the main metabolic potential found in each evaluated MAG Section 2, Considerations for DNA‐SIP Supplementary Fig. 1. Above: Measured chemical parameters from the water column in μM; DOC: dissolved organic carbon, DIC: dissolved inorganic carbon, TN: total nitrogen. Below: CTD profiles across the water column. Supplementary Fig. 2. Diagram of workflow from incubations to DNA‐SIP experiments Supplementary Figs 3–8. Phylogenetic tree showing the 16S rRNA of active OTUs by phyla and a closest neighbouring 16S rRNA gene obtained from a publicly available genome closely related to the MAG of that phyla (based on 34 single‐copy marker genes) is shown. Supplementary Table 5. List of 34 single‐copy marker genes used for phylogenetic analysis of MAGs Supplementary Fig. 9. Chemical measurements from incubations at 72 h Supplementary Table 6. Recipe for MDV media used for culturing the diatom biomass Supplementary Figs 10–13. Reconstructed metabolic pathways of selected MAGs [file EMI-23-2709-s002.docx]

Supplementary file 1

**A diverse uncultivated microbial community is responsible for organic matter degradation in the Black Sea sulfidic zone**

Saara Suominen^1*^, Nina Dombrowski^1^, Jaap Sinninghe Damsté^1,2^, and Laura Villanueva^1^

^1^Department of Marine Microbiology and Biogeochemistry, NIOZ Royal Netherlands Institute for Sea Research and Utrecht University, The Netherlands

^2^Department of Earth Sciences, Faculty of Geosciences, Utrecht University, The Netherlands.

* Corresponding author, [saara.suominen@nioz.nl](mailto:sigrid.van.grinsven@nioz.nl)

Postal address: NIOZ, PO Box 59, 1790 AB Den Burg (Texel), The Netherlands

Telephone & fax: Telephone: +31 (0)222 369 504 Fax: +31 (0)222 319 674

**Contents:**

**Section 1,** A description of the main metabolic potential found in each evaluated MAG

**Section 2,** Considerations for DNA-SIP

**Supplementary Figure 1.** Measured chemical parameters from the water column

**Supplementary Figure 2.** Diagram of workflow from incubations to DNA-SIP experiments

**Supplementary Figures 3-8.** Phylogenetic tree showing the 16S rRNA of active OTUs by phyla and a closest neighbouring 16S rRNA gene obtained from a publicly available genome closely related to the MAG of that phyla (based on 34 single-copy marker genes) is shown.

**Supplementary Table 5.** List of 34 single-copy marker genes used for phylogenetic analysis of MAGs

**Supplementary Figure 9.** Chemical measurements from incubations at 72 hours

**Supplementary Table 6.** Recipe for MDV media used for culturing the diatom biomass

**Supplementary Figures 10-13.** Reconstructed metabolic pathways of selected MAGs

**Section 1.**

**Description of the individual MAGs**

In the following section we shortly describe the metabolic potential of the individual MAGs and show a possible cell model for the most common community members. A phylogenetic tree is shown built from 16S rRNA gene diversity of the active OTUs of the incubation experiments (bold), and the closest relative 16S rRNA gene available to the analysed MAGs (Supplementary Figures 3-8). These were found by first making a phylogenetic tree with single-copy marker genes with all publicly available genomes of the same taxonomic group, and finding the closest relative with a 16S rRNA gene sequence. This was then used to estimate their position in relation to the active OTUs.

*Marinimicrobia*

*Marinimicrobia* (previously known as Marine Group A or SAR406) is commonly found throughout marine environments, but is an especially dominant microbial group in oxygen minimum zones like the Black Sea (Hawley et al. 2017). We assess the metabolic properties of the *Marinimicrobia* MAG (bin1) assembled from our study site in 2013 by comparing the full pathways found in the genetic repertoire (Supplementary Figure 2). Previous genomic data suggest this common marine phylum can use diverse alternative electron acceptors, with genes like polysulfide reductase, nitrate reductase and nitrous oxide reductase (Wright et al. 2014, Hawley et al. 2017, Plominsky et al. 2018). Instead of the Nar complex for dissimilatory nitrate reduction, our MAG encodes a periplasmic nitrate reductase (napAGH) located in the same contig with cytochrome c reductases (petBC). No nitrite reductases were found, neither dissimilatory nor assimilatory, unlike results from previous OMZ MAGs (Bertagnolli et al. 2017). In addition no dissimilatory polysulfide reductases were identified, though this has been one of the major connecting factors for marine Marinimicrobia genomes previously (Hawley et al. 2017). Like other publicly available *Marinimicrobia* genomes (Bertagnolli et al. 2017) we however find the partial gene complex associated with the oxidation of carbon monoxide (*coxM, and coxS*). Recent MAGs from oxygen minimum zones and the hypoxic northern Gulf of Mexico suggested also a niche for degradation of macromolecular organic matter and peptides (Bertagnolli et al. 2017, Thrash et al. 2017). A possible peptide-degrading MAG retrieved from a methanogenic bioreactor has been found to be reliant on hydrogen and electron-confurcating hydrogenases for its energetic processes (Nobu et al. 2013, Hawley et al. 2017). Our MAG has many similarities with electron transferring (*RnfA-E*) and electron bifurcating complexes (*MvhADG/HdrABC*) as well as peptidases, peptide transporters (*ddpB-D, OPT*), and some of the amino acid degradation pathways as were detected in the peptide-degrading MAG. The end products of these degradation pathways can be directed to central metabolism and synthesis of biomolecules through the full TCA-cycle, or possibly to the production of carboxylic acids through the use of acetyl-CoA synthetase (*acs*). The main difference to the other MAGs that we compare it to is that there are more genes capable of NADH reoxidation through membrane-associated complexes. It encodes for NADH-quinone oxidoreductases (*nuoA*-*N*) as well as a sodium-translocating NADH-quinone oxidoreductases (*nqrA-D,F*) giving it the possibility to use diverse types of oxidative phosphorylation for energy capture. Overall, a combination of genetic capabilities partly known from Marinimicrobia in oxygen minimum zones and partly from a more reduced environment points to an organism that is highly adapted to the conditions in the sulfidic zone of the Black Sea and is capable of exploiting extant organic matter despite thermodynamically unfavourable conditions for the degradation processes.

*Chloroflexi*

The second of the most abundant phyla in our amplicon dataset belonged to the *Chloroflexi* phylum, further classified into classes *Dehalococcoidia and Anaerolineae* (bin31, bin47, bin52). This phylum has recently been linked to carbon degradation in diverse environments ranging from the deep pelagic ocean (Landry et al. 2017) to sediment environments (Hug et al. 2013). Sedimentary single amplified genomes (SAGs) have found also other interesting similarities between the metabolisms of organisms related to the *Chloroflexi*. Usually a diverse range of ABC transporters, presence of the Wood-Ljungdahl pathway and a lack of respiratory genes points to an obligately fermentative, organotrophic, and possibly acetogenic lifestyle (Kaster et al. 2014, Wasmund et al. 2014, Fullerton et al. 2016, Sewell et al. 2017). Likewise, our *Chloroflexi* MAGs had by far the most CAZymes, extracellular peptidases and ABC transporters of the analysed MAGs, possibly allowing them to access diverse organic carbon sources from their surroundings (Figure 4 and Figure 5). A full Wood-Ljungdahl pathway of acetogenesis was also characterized in our most complete MAG (bin31). Conversely our third *Chloroflexi* MAG (bin47) is related to the class *Dehalococcoidia* and shows a very different genetic assemblage. The *Dehalococcoidia* have been defined by their capabilities for dehalogenation as a terminal reduction pathway, though recently genomes retrieved from the deep biosphere have been classified as anaerobic acetogens capable of fermentation of plant polymers as well as amino acids and organosulphur compounds (Hug et al. 2013, Wasmund et al. 2014). In our *Dehaloccoidia* bin, we did not find indications of complex substrate degradation, reductive dehalogenation or the Wood-Ljungdhal pathway, but several copies of genes for the beta-oxidation of fatty acids (*fadD*, *fabG*), for formate and hydrogen utilisation and propionate degradation. Like previous studies, the gene repertoire of the *Chloroflexi* MAG belonging to the order *Anaerolineales* (bin31, bin52) suggests a metabolically diverse heterotroph, with a collection of substrate scavenging genes, while the *Dehalococcoidia* (bin47) encoded for more genes indicative of syntrophic degradation of intermediate substrates.

*Cloacimonetes*

The *Cloacimonetes* phyla that is the third most common phylum in the Black Sea at 1000 m water depth, has been previously predominantly described from methanogenic digesters (Pelletier et al. 2007, Nobu et al. 2015, Stolze et al. 2016, Dyksma et al. 2019). Amino acid (Pelletier et al. 2007, Stolze et al. 2016) as well as cellulose degradation (Limam et al. 2014) have been suggested as the main carbon acquisition methods of this organism group. Our *Cloacimonetes* MAG (bin17) shows also potential for the degradation of amino acids. It encodes diverse extracellular peptidases as well as pathways for the degradation of histidine, arginine, asparagine, lysine and the glycine cleavage pathway. Previously *Cloacimonetes* sp. has been characterized to act as a syntrophic propionate degrader (Nobu et al. 2015, Dyksma et al. 2019). Contrastingly, we did not find the full set of genes necessary for propionate degradation, in fact only propionyl-CoA carboxylase was detected (*pccB*). In addition, we did not detect the complete gene set of glycolysis or the TCA cycle, though the TCA cycle intermediates could be linked together through pyruvate. However, bin17 did contain the characteristic electron transferring complexes and hydrogenases (Rnf, MvhHdr, FixAB), as well as a Na+ H+ antiporter (mnhA-G), for building a membrane potential and syntrophic metabolism (Dyksma et al. 2019). *Cloacimonetes* bin17 possibly is an obligate fermenter utilizing diverse organic matter and producing acetate while conserving energy through electron bifurcating complexes and ferredoxin.

*Deltaproteobacteria*

Two MAGs were recovered from the *Deltaproteobacteria* class that belonged to two main known sulfate-reducing genera, *Desulfatiglans* from the *Desulfurellales* class, and *Desulfobulbacaea* from *Desulfobacterales.* All cultured representatives of *Desulfatiglans* grow on aromatic hydrocarbons, but the genus has recently been described with capabilities to oxidize diverse organic substrates, sulfatases as well as hydrogen (Jochum et al. 2018). The MAG affiliated to Desulfatiglans (bin40) contained the *dsrAB* genes for dissimilatory sulfite reduction. Interestingly, it encoded over 10 copies of branched chain amino acid ABC transporter genes as well as a formate dehydrogenase. It contained the full pathway for fatty acid degradation, leading to the hypothesis that it could act as a syntrophic fatty acid degrader for products resulting from the fermentation of branched chain amino acids (Schink and Stams 2013, Narihiro et al. 2016). In addition it also contains genes necessary for the utilisation of propanoate and butanoate, similarly an important substrate for syntrophic degraders. Our *Desulfobulbacaea* MAG (bin81) contained *dsrAB* as well as a nitrite reductase gene (*nirB*). It was our only MAG with genes for flagellar motility. In fact this MAG was not abundant at 1000 m depth, which can explain the genetic makeup more suitable for surface waters with dynamic redox cycling, with cytochromes and a full *Nuo* complex I gene collection. We also did not find the typical hydrogenases and electron transfer complexes (other than *fixAB*), as in all other MAGs. However, this MAG did contain a propanoate conversion pathway, as well as the branched-chain amino acid transporters and degradation pathways similar to the *Desulfatiglans* MAG, making it possible that it occupies a similar niche in less reduced conditions.

*Omnitrophica*

Very little is known of the phyla *Omnitrophica* previously called candidate division OP3, which is part of the PVC superphylum (Glockner et al. 2010). Members of this phyla have been identified as magnetotactic sulfur-oxidizers (Kolinko et al. 2016), with nitrate reductase and methanotrophic genes (Momper et al. 2017) and possessing Fe,Fe-hydrogenase genes for producing H_2_ during fermentation (Dombrowski et al. 2017). Visualization of cells from limonene-degrading methanogenic reactors found small round cells mainly attached to larger ones (Rotaru et al. 2012). A closed genome of the phyla was characterized as an ultrasmall predator, living off of polymers produced by an archaeal prey cell (Kizina 2017). Our *Omnitrophica* MAG (bin146) had a small genome and also was markedly low in abundance in the 2013 amplicon dataset (filter pore size 0.7 µm) compared to 2017 (filter pore size 0.2 µm) possibly indicating a small cell size that escaped the sampling in 2013. This MAG was the least complete of our analysed genomes (56%), making it difficult to infer the presence of full degradation pathways. However, we did find partial pathways for carbon degradation through glycolysis to acetate, as well as few genes for the *Nuo* NADH oxidoreductase, the antiporter *mnh*, both F- and V-type ATP synthases and a pyrophosphatase. We found only one extracellular peptidase, though the transport of oligopeptides through the membrane seems possible with the transporters *ddpB* and ABC.PE.A1. We found evidence for hydrogen production with formate hydrogenlyase, and electron transfer with *fixAB*. Similar to Kizina et al. (Chapter 5 in dissertation, 2017) we find genes for the type II secretion / Type IV pilus assembly system possibly linked to an attachment to a host cell. Possibly this MAG performs hydrogen and acetate producing fermentations by scavenging polymers from its environment or a host cell.

*Woesearchaeota*

The *Woesearchaeota* are a group belonging to the recently described DPANN branch of archaea, and is found worldwide in diverse habitats, with a preference for anoxic environments. Like most DPANN, the *Woesearchaeota* genomes analysed so far lack essential cellular metabolisms, pointing to nutritional dependencies with other organisms (Castelle et al. 2015, 2018, Liu et al. 2018, Dombrowski et al. 2019). The *Woesearchaeota* MAGs retrieved from the study site have relatively few genes from CAZymes and few extracellular peptidases. Similar to previous studies on DPANN genomes, the two *Woesearcheota* MAGs had small genomes (1.46 Mbp). Intriguingly bin 61 encoded for a partial TCA cycle as well as amino acid transporters, which are often absent in *Woesearchaeota* and DPANN in general. The main common features between the archaeal bins were an almost complete glycolysis pathway and a pentose phosphate pathway, flagellar genes and genes for an archaeal proteasome. In addition a considerably larger percentage of genes were assigned hypothetical (mean 43% for bacteria, 58% for archaea), indicating a largely unknown genetic composition of these organisms.

**Section 2.**

**Considerations for DNA-SIP**

We chose a relatively short period of time for the SIP incubations (72 hours), and locally relevant levels of substrate addition (approximately 1.3 mg/l) in an attempt to not excessively change the environmental conditions in-situ. The simple experimental design and objective of catching initial organic carbon compound utilizers, meant that we were unable to chemically follow the degradation of substrate in our experiments (Supplementary figure 9). Similar to Orsi et al. (2016), our objective was to detect primary degraders of organic matter pools. As microbial community members are highly connected with each other in anoxic degradation processes, a longer incubation time might result in a labelling of organisms utilising secondary degradation products, and a dilution of the label throughout the community. Additionally, a comparable experimental design in a previous expedition showed labelling of bacterial lipids with a 4 day incubation time (unpublished data). The distribution of relative copy numbers of individual OTUs across the density gradient shows that generally a shift in 16S rRNA gene density is found in the OTUs determined as active (Figure 2, main text), but the level of random noise in our experiment cannot be quantified. We also do not consider our MWE values as absolute but more as a comparable way to determine the level of shift in buoyant density across taxonomic groups. To fully confirm the incorporation of substrate to individual cells, more tests with techniques like RAMAN spectroscopy and nanoSIMS would be necessary, and will require development and standardization of FISH probes for uncultured organism groups.

**
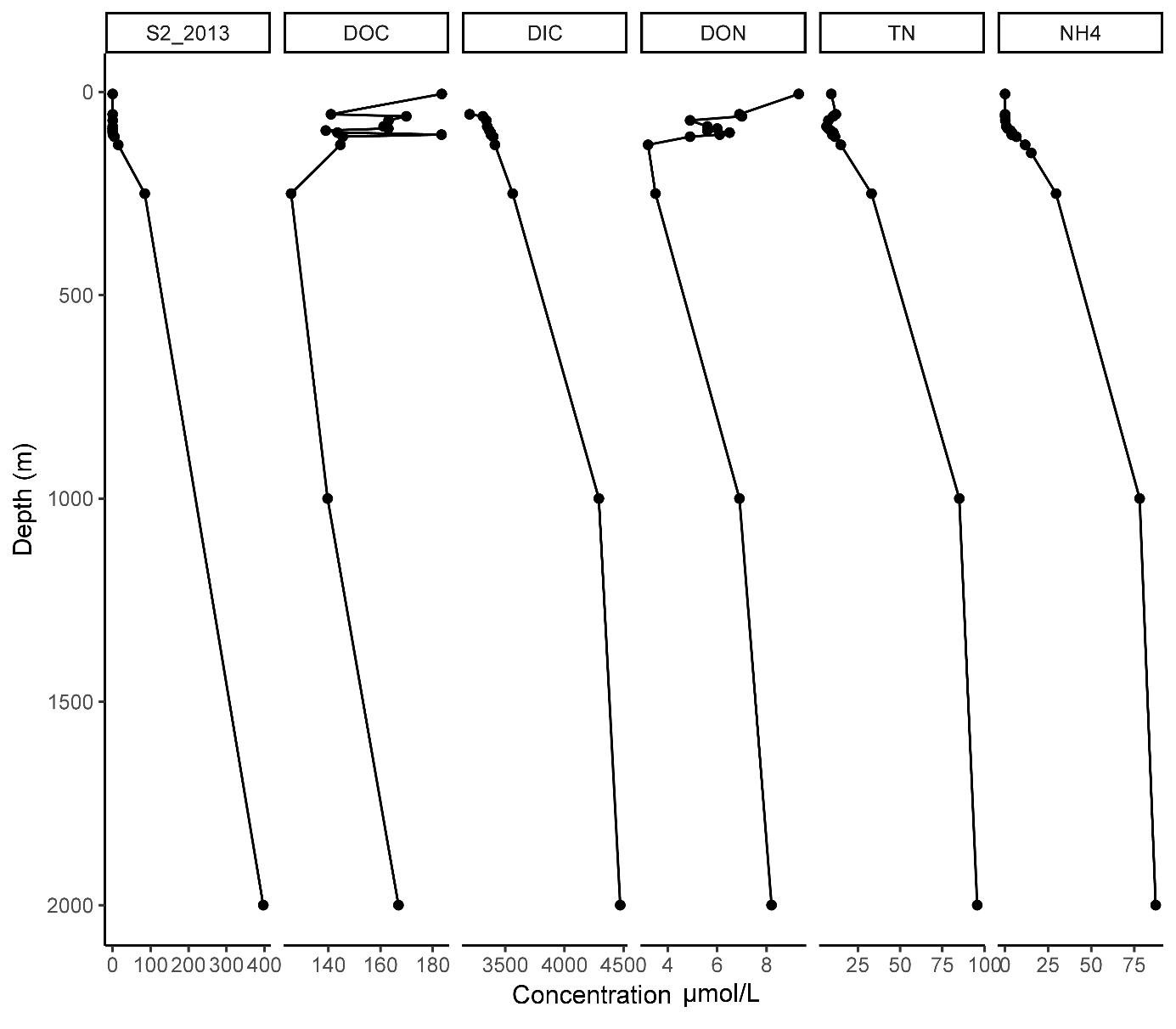
**

**Supplementary Figure 1,** Measured chemical parameters from the water column in µM; DOC: dissolved organic carbon, DIC: dissolved inorganic carbon, TN: total nitrogen.

**Supplementary Figure 3.** Diagram of experimental procedure for the DNA-SIP incubations

**Supplementary Figure 3,** A phylogenetic tree showing the 16S rRNA gene diversity of active OTUs (in bold) and the MAG belonging to the phyla Marinimicrobia (in red). In addition a closest neighbouring 16S rRNA gene obtained from a publicly available genome closely related to bin1 (based on 34 single-copy marker genes) is shown. Accession number, taxonomy and isolation source are shown for sequences retrieved from SILVA database release 128. Bootstrap values from Ultrafast Bootstrap estimation in IQtree.

**Supplementary Figure 4,** A phylogenetic tree showing the 16S rRNA gene diversity of active OTUs (in bold) belonging to the phylum *Chloroflexi*. In addition a closest neighbouring 16S rRNA gene obtained from a publicly available genomes closely related to bin47, bin52 and bin31 (based on 40 single-copy marker genes) is shown (in red). Accession number, taxonomy and isolation source are shown for sequences retrieved from SILVA database release 128.

**Supplementary Figure 5,** A phylogenetic tree showing the 16S rRNA gene diversity of active OTUs (in bold) belonging to the phylum *Cloacimonetes*. In addition a closest neighbouring 16S rRNA gene obtained from a publicly available genome closely related to bin17 (based on 40 single-copy marker genes) is shown (in red). Accession number, taxonomy and isolation source are shown for sequences retrieved from SILVA database release 128.

**Supplementary Figure 6,** A phylogenetic tree showing the 16S rRNA gene diversity of active OTUs (in bold) belonging to the phylum *Omnitrophica*. In addition a closest neighbouring 16S rRNA gene obtained from a publicly available genome closely related to bin146 (based on 40 single-copy marker genes) is shown (in red). Accession number, taxonomy and isolation source are shown for sequences retrieved from SILVA database release 128.

**Supplementary Figure 7,** A phylogenetic tree showing the 16S rRNA gene diversity of active OTUs (in bold) belonging to the *Deltaproteobacteria*. In addition a closest neighbouring 16S rRNA gene obtained from a publicly available genome closely related to bin40 and bin81 (based on 34 single-copy marker genes) is shown (in red). Accession number, taxonomy and isolation source are shown for sequences retrieved from SILVA database release 128.

**Supplementary Figure 8,** A phylogenetic tree showing the 16S rRNA gene diversity of active OTUs belonging to the *Woesearchaeota.* In addition a closest neighbouring 16S rRNA gene obtained from a publicly available genome closely related to bin111 and bin61 (based on 34 single-copy marker genes) is shown (in red). Accession number, taxonomy and isolation source are shown for sequences retrieved from SILVA database release 128.

**Supplementary Table 5.** List of 34 single-copy marker genes used for phylogenetic analysis of MAGs

DNGNGWU00001

DNGNGWU00002

DNGNGWU00003

DNGNGWU00005

DNGNGWU00006

DNGNGWU00007

DNGNGWU00009

DNGNGWU00010

DNGNGWU00011

DNGNGWU00012

DNGNGWU00014

DNGNGWU00015

DNGNGWU00016

DNGNGWU00017

DNGNGWU00018

DNGNGWU00019

DNGNGWU00021

DNGNGWU00022

DNGNGWU00023

DNGNGWU00024

DNGNGWU00025

DNGNGWU00026

DNGNGWU00027

DNGNGWU00028

DNGNGWU00029

DNGNGWU00030

DNGNGWU00031

DNGNGWU00032

DNGNGWU00033

DNGNGWU00034

DNGNGWU00036

DNGNGWU00037

DNGNGWU00039

DNGNGWU00040

**Supplementary figure 9.** Chemical measurements from incubations at 72 hours as µmol/l, except cells/µl which is flow cytometry measurements of cell counts. Red dots are from labelled incubations and blue dots are from unlabelled incubations. DIC: dissolved inorganic carbon, TN: total nitrogen, DOC: dissolved organic carbon, DON: dissolved organic nitrogen. Measurements are from the incubations used for qSIP analysis (50 L) and two replicate incubations used for chemical measurements only, with a total volume of 20 L. Control incubations had no addition of substrate (volume 20L).

**Supplementary Table 5.** Recipe for MDV media for diatom cultures

| **1. Mineral mix** | **Stock solution (g/l)** | **Quantity (ml Stock/ L media)** | **Molarity (mM)** |
| --- | --- | --- | --- |
|  |  |  |  |
| NaCl | 241 | 100 | 400 |
| MgCl_2_ · 6H_2_O | 435 | 20 | 43 |
| KCl | 54 | 10 | 7,2 |
| Na_2_SO_4_ | 32 | 100 | 23 |
| CaCl_2_ · 2H_2_O | 160 | 10 | 11 |
|  |  |  |  |
| **2. Autoclave** |  |  |  |
| **3. After cooling add the following filter sterilized components** | | | |
|  |  |  |  |
| NaNO_3_ | 100 | 5 | 6 |
| NaHCO_3_ | 18 | 10 | 2 |
| NaH_2_PO_4_ · H_2_O | 6,9 | 1 | 0,05 |
| Na_2_SiO_3_ · 5H_2_O | 15,9 | 2 | 0,15 |
| Citrate mix |  | 10 |  |
| Trace metal mix |  | 1 |  |
| Vitamins 8 mix |  | 1 |  |
| M2 |  | 1 |  |
|  |  |  |  |
| ****for labelled media replaced unlabelled stock solution*** | | | |
| *Na^15^NO_3_* | *15* | *5* |  |
| *NaNO_3_* | *85* | *5* |  |
| ****for labelled media replaced unlabelled stock solution*** | | | |
| *NaH^13^CO_3_* | *5,4* | *10* |  |
| *NaHCO_3_* | *12,6* | *10* |  |
|  |  |  |  |
| **A. Citrate mix** | **Quantity (g/L)** | |  |
| C_6_H_8_O_7_ | 0,27 |  |  |
| Fe-NH_4_-citrate | 0,36 |  |  |
|  |  |  |  |
| **B. Trace Metal mix** | **Stocks (g/l)** | **ml stock / L mix** |  |
| CuSO_4_ · 5H_2_O | 9,8 | 1 |  |
| ZnSO_4_ · 7H_2_O | 22 | 1 |  |
| CoCl_2_ · 6H_2_O | 10 | 1 |  |
| MnCl_2_ · 4H_2_O | 18 | 1 |  |
| Na_2_MoO_4_ · H_2_O | 5,8 | 1 |  |
| Na_2_SeO_3_ · H_2_O | 0,011 | 0,1 |  |
|  |  |  |  |
| **C. Vitamins 8 mix** | **Stocks (g/ 100 ml)** | **ml stock / 100 ml mix** |  |
| Biotin* | 0,004 | 0,1 |  |
| Thiamine-HCl | 0,02 | 10 |  |
| Cyanocobalamin | 0,08 | 0,1 |  |
| Folic acid* | 0,008 | 0,1 |  |
| Inositol | 0,02 | 1 |  |
| Nicotinic acid | 0,04 | 1 |  |
| Thymine* | 0,012 | 1 |  |
| Ca-d-pantothenate | 0,04 | 1 |  |
| **Dissolve first in 1N NaOH and then bring to volume with mQ water* | | | |
|  |  |  |  |
| **D. M2** | **Quantity (g/L)** | |  |
| KBr | 39 |  |  |
| SrCl_2_ · 6H_2_O | 10 |  |  |
| AlCl_3_ · 6H_2_O | 0,014 |  |  |
| LiCl | 0,003 |  |  |
| KBr | 0,01 |  |  |
| H_3_BO_3_ | 11 |  |  |
| RbCl | 0,03 |  |  |

***
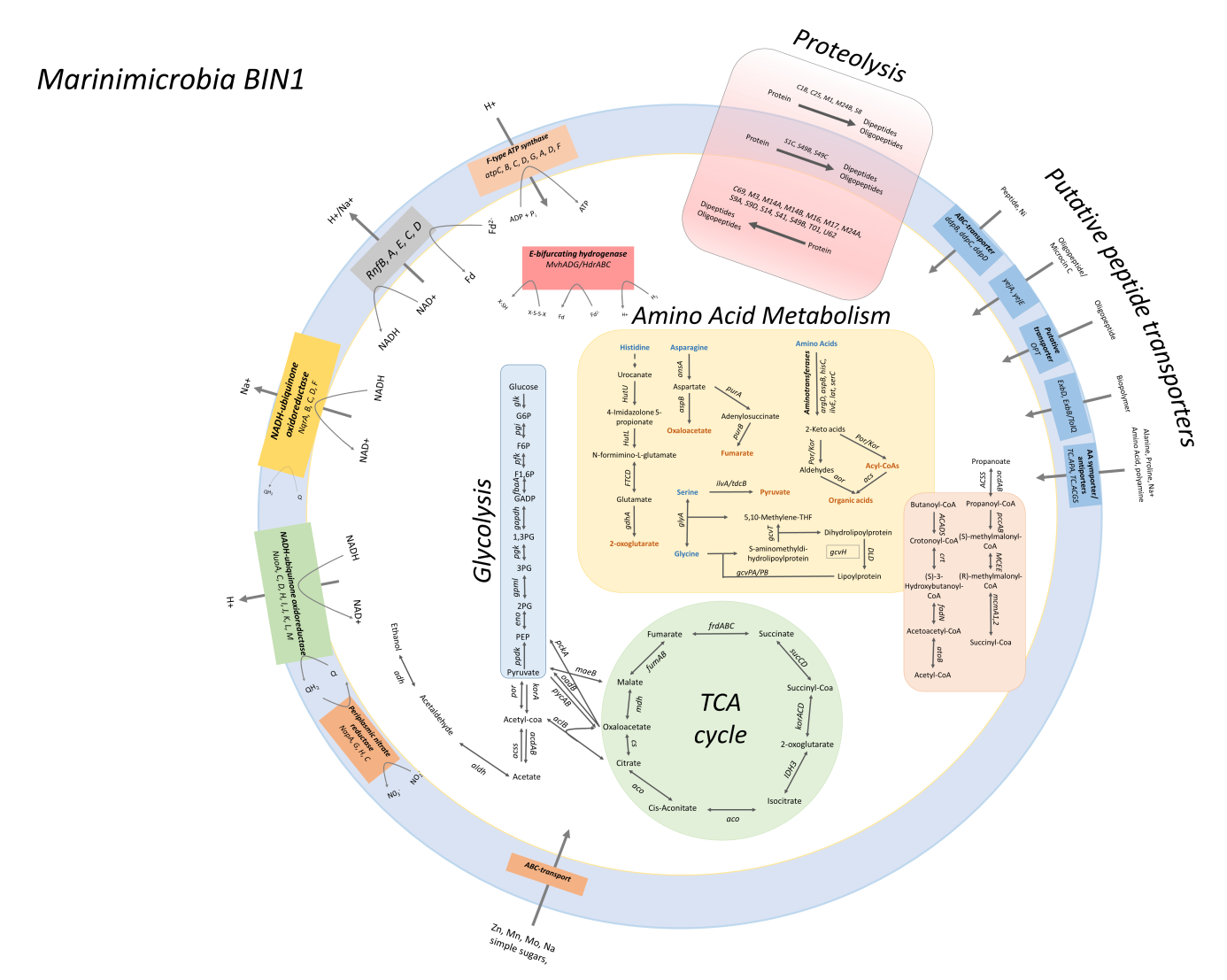
***

**Supplementary Figure 10,** Reconstructed metabolic pathways of bin1

**
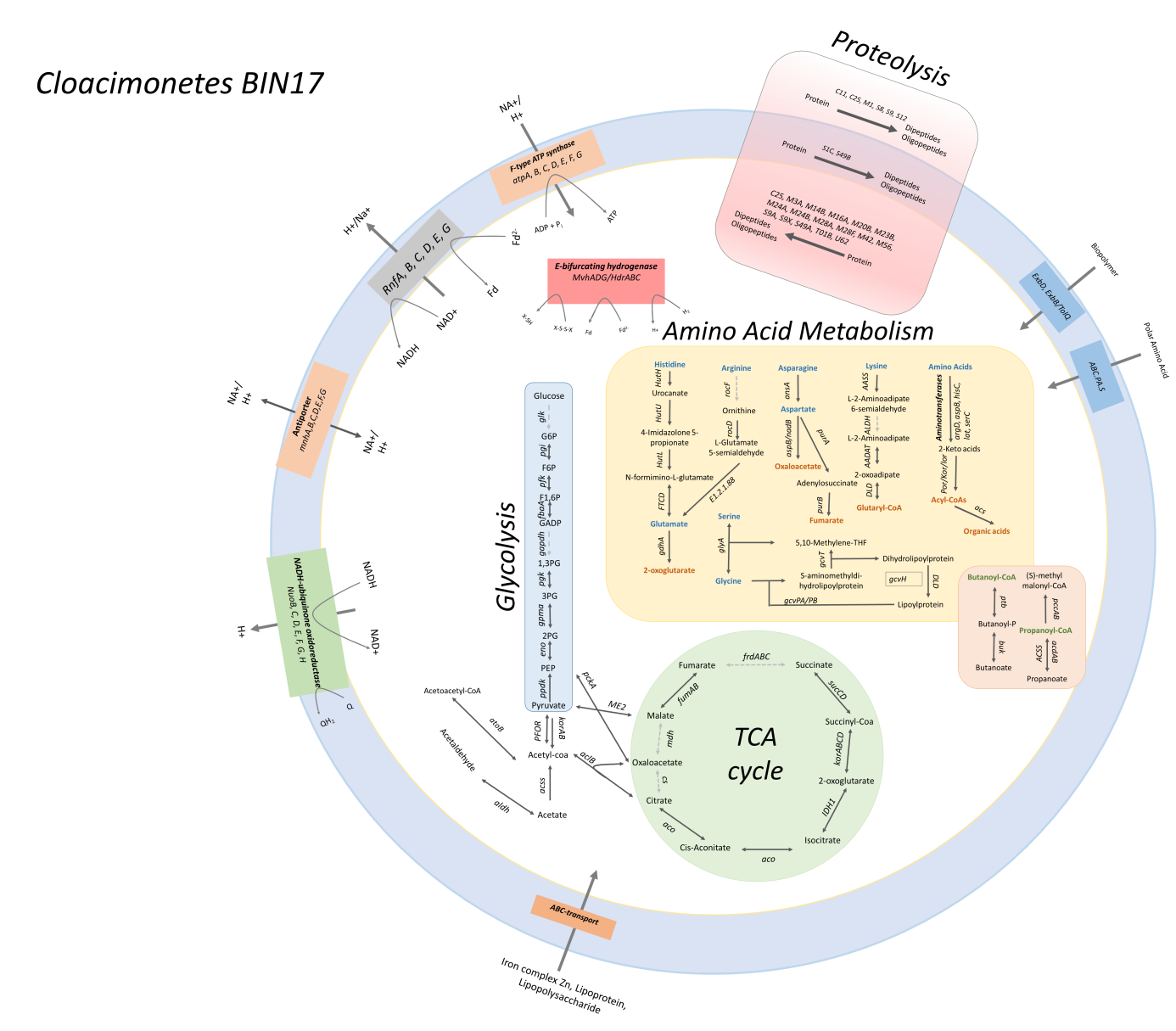
**

**Supplementary Figure 11,** Reconstructed metabolic pathways of bin17

**
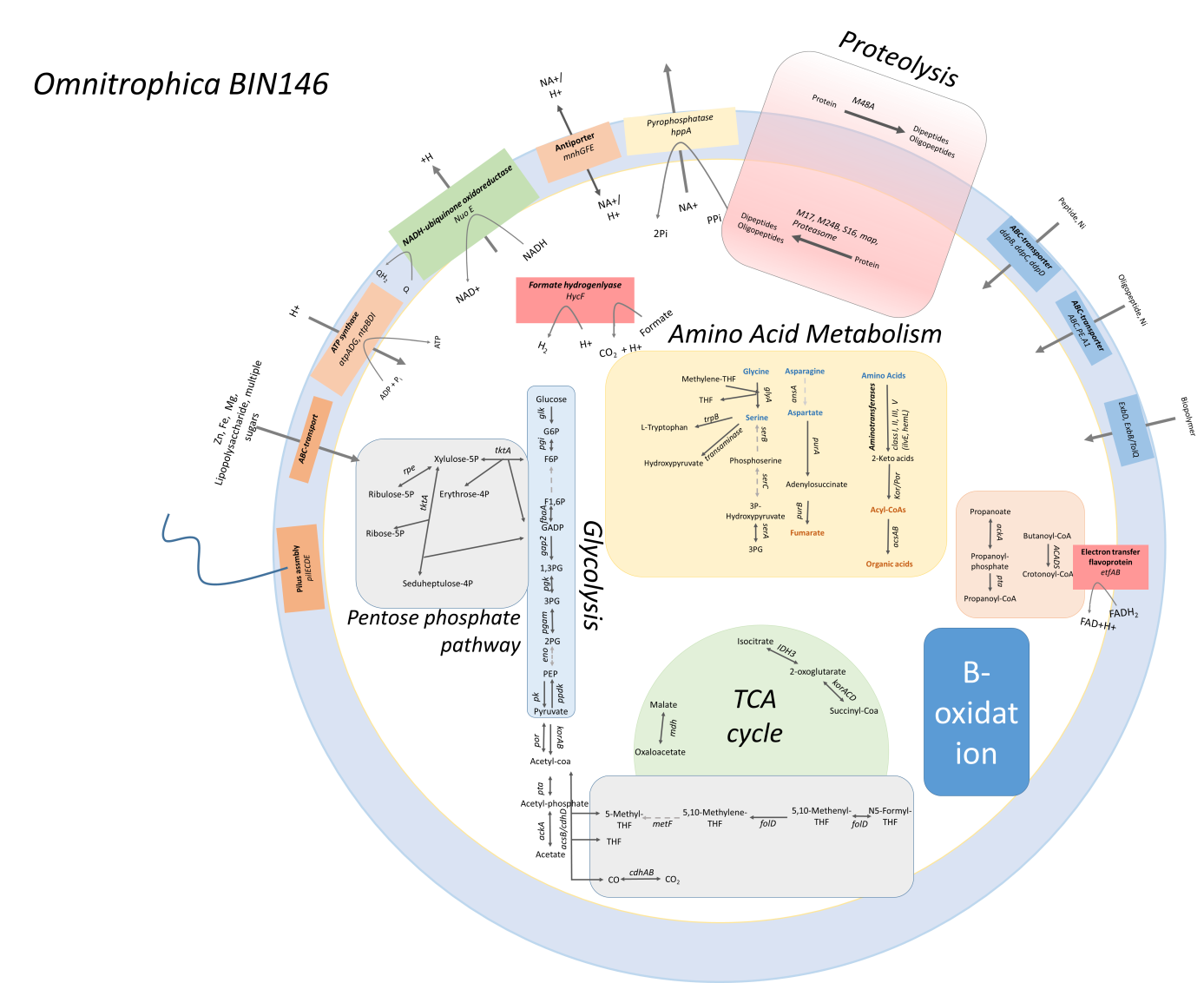
**

**Supplementary Figure 12,** Reconstructed metabolic pathways of bin146

**
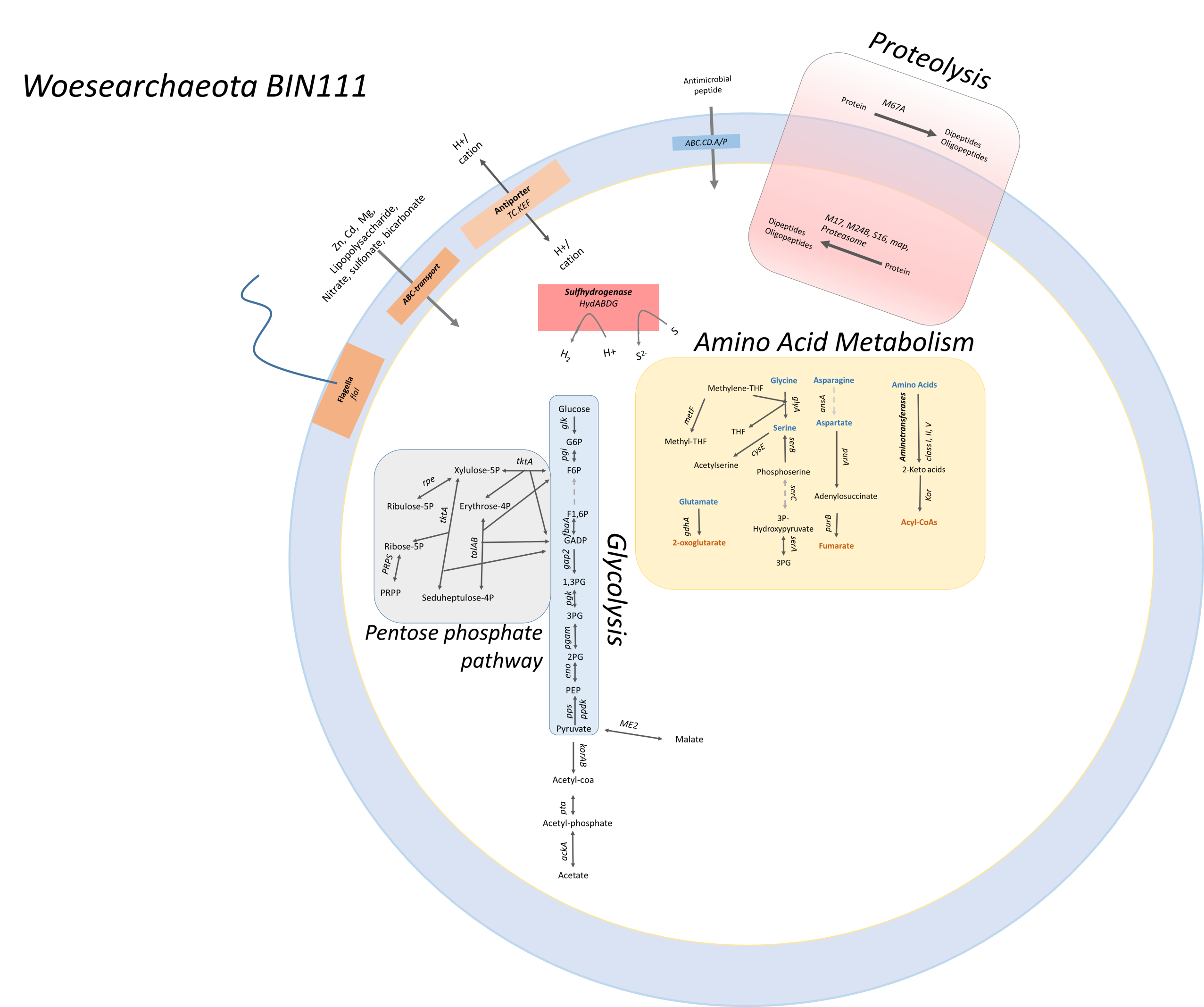
**

**
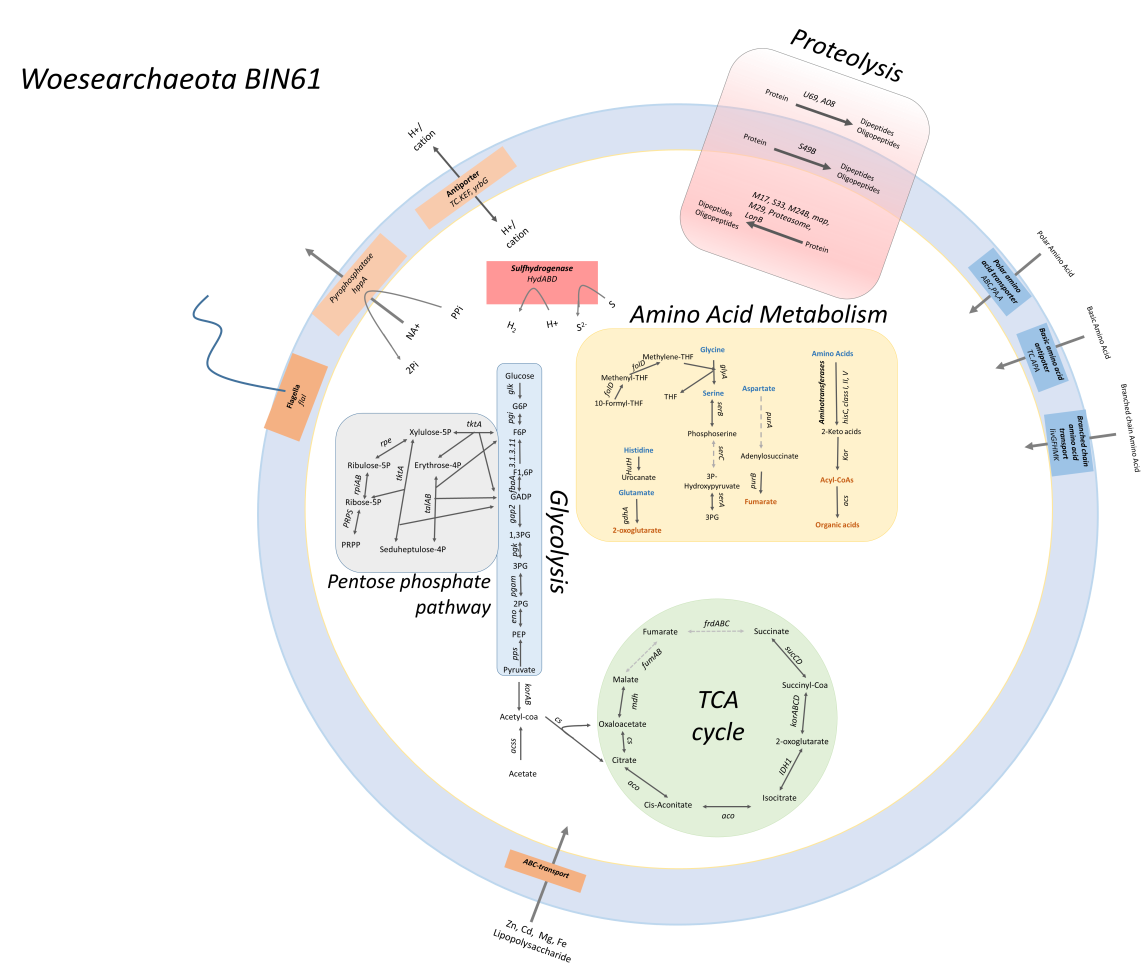
**

**Supplementary Figure 13,** Reconstructed metabolic pathways of the bins belonging to the phylum *Woesearcheota* (bin111 and bin61).
